# Isolating Al Surface Sites in Amorphous Silica–Alumina by Homogeneous Deposition of Al^3+^ on SiO_2_ Nanoparticles

**DOI:** 10.1021/acsanm.4c04544

**Published:** 2024-10-31

**Authors:** Ferdy Coumans, Brahim Mezari, Norwin Zuidema, Jason M. J. J. Heinrichs, Emiel J. M. Hensen

**Affiliations:** Laboratory of Inorganic Materials and Catalysis, Department of Chemical Engineering and Chemistry, Eindhoven University of Technology, PO Box 513, Eindhoven, MB 5600, the Netherlands

**Keywords:** amorphous silica−alumina, homogeneous deposition-precipitation, SiO_2_ nanoparticles, Brønsted acid sites, catalytic pyrolysis, thermogravimetric analysis

## Abstract

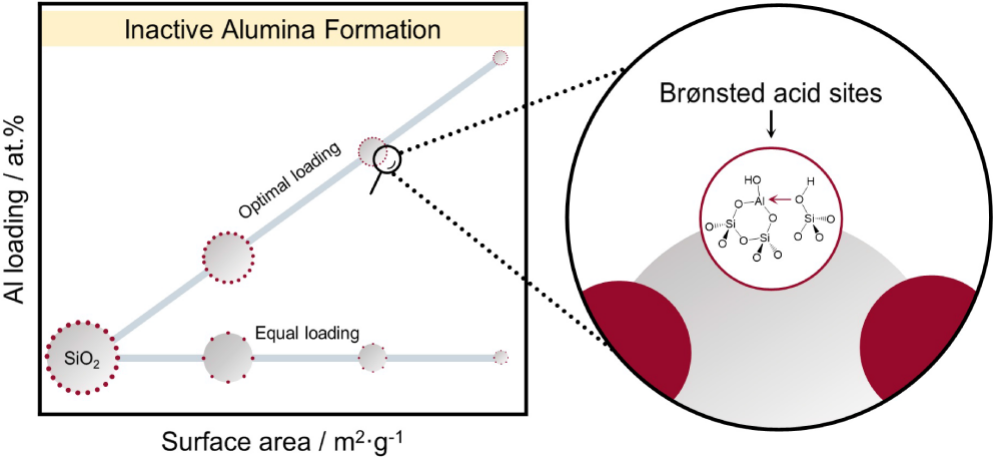

Well-defined amorphous silica–alumina (ASA) with a relatively low Al loading were synthesized by homogeneous deposition-precipitation of Al^3+^ on SiO_2_ nanoparticles to understand the nature and formation of Brønsted acid sites (BAS). The amount of Al grafted relative to the silanol density was varied by variation of the size of SiO_2_ nanoparticles, reflected by their surface areas between 90 and 380 m^2^·g^–1^. Two sets of ASA were synthesized, one aiming at a SiOH/Al ratio of 3, corresponding to the maximum amount of BAS represented by Al^3+^ perturbation of SiOH groups, and the second one aimed at studying the impact of Al dispersion by using a constant Al loading (Si/Al ≈ 103). ^27^Al MAS NMR spectroscopy confirmed that the first sample set only contained tetrahedral Al species. Calcination did not affect the Al coordination. CO IR spectroscopy revealed that the BAS concentration substantially varied in the 15–133 μmol·g^–1^ range by varying the Al loading and the SiO_2_ nanoparticle size. At equal Al loading, the BAS concentration increased from 15 to 46 μmol·g^–1^ with increasing SiO_2_ surface area. Less than 30% of all grafted Al sites gave rise to BAS, independent of the surface area and calcination temperature. The ASA samples were screened for their catalytic performance in pyrolytic cracking of ultrahigh molecular weight polyethylene in a thermogravimetric analysis apparatus. The performance in pyrolysis, as gauged by the temperature at which the weight loss rate was highest, increased with the Brønsted acidity. The cracking temperature decreased from 490 °C without a catalyst to 463 °C using the most acidic ASA. At equal Al loading, the pyrolysis temperature decreased with increasing surface area, indicating that, besides acidity, cracking also benefits from a higher surface area where the long polymer chains can adsorb. Compared to zeolite, ASA produced more liquid hydrocarbons and less coke.

## Introduction

Over the past 100 years, humanity has witnessed immense growth in overall wealth and well-being. This economic growth has been fueled by abundant and cheap fossil feedstock used to manufacture fuels and base chemicals. Base chemicals, such as olefins and aromatics, are widely employed as precursors to monomers for various plastics with beneficial functional properties. About 17% of fossil carbon feedstock is converted into base chemicals. Given the expected lower demand for liquid transportation fuels, the fraction of chemicals derived from petroleum in oil refineries will likely increase. The petrochemical industry accounts for about 5% of the total CO_2_ emissions, which will increase due to global economic growth.^1^ The urgency to reduce CO_2_ emissions due to concerns about climate change will lead to feedstock diversification. While renewable carbon sources include biomass and CO_2_ captured from the air, recycling waste such as plastics is another approach to closing the carbon cycle.^2,3^

Currently, most used plastics end up in landfills or the environment (∼79%). Only a small fraction is recycled (∼9%), and the rest is incinerated (∼12%).^4^ Given the growing demand for plastics, it is reasonable to expect more plastic waste to be available, outpacing efforts to reduce plastic use.^5^ Therefore, using spent plastics as a feedstock might not only reduce CO_2_ emissions but also mitigate the buildup of plastics in the environment. Among the many mechanical and chemical recycling processes, pyrolysis is a promising approach to convert plastics, such as polyolefins, which are hard to depolymerize or repurpose via other means, into valuable new chemicals.^6−10^ It is especially versatile for processing heterogeneous and contaminated plastic mixtures. Pyrolysis takes place at mild to high temperatures in the absence of oxygen.^6,9,10^ The reaction mechanism depends on the mode of operation, which includes thermal and thermocatalytic pyrolysis. The former process proceeds via free-radical depolymerization, while the latter occurs mainly through a carbocation mechanism involving acid catalysts.^8−10^ Subsequent steps involve hydrogen transfer and β-scission, and their relative rates depend on the reaction conditions. Adding a catalyst can lower the pyrolysis temperature and provide a more favorable product distribution. However, poor accessibility of the molten polymers to the active sites can limit the catalytic performance.^11^ It has also been shown that a high melt viscosity, particularly associated with high molecular weight polymers, limits the complete utilization of active sites in porous catalyst particles.^12^ Furthermore, catalyst recovery and deposition of inorganic and carbonaceous compounds typically hamper the reusability of the catalyst.^10,13^

Solid acids like zeolite, ordered mesoporous aluminosilicates, clays, and silica–alumina are typical catalysts for the pyrolysis of plastics.^9^ Zeolites such as HZSM-5, HY, HBeta, and HMOR have been extensively investigated for catalytic pyrolysis of plastic waste.^13,14^ The overall performance typically depends on the acid site concentration and pore structure.^15^ Pore size, connectivity, and dimensionality influence the activity and the product distribution. The cracking of the long-chain hydrocarbons in plastic waste starts on acid sites at the external surface of zeolites, as diffusion of such large molecules into the micropores is strongly hindered. Using hierarchical zeolites with substantial mesoporosity, which effectively increases the external surface area, can help improve the catalytic performance and stability.^13,16^ Amorphous silica–alumina (ASA) catalysts are usually less active than zeolites due to their weaker acidity.^17,18^ However, higher liquid product yields are typically reported for ASA, making it more attractive and cost-effective than zeolite.^18−21^ ASAs are solid acids widely used in industrial processes such as hydrocracking, isomerization, and alkylation.^22,23^ These materials are characterized by weak-to-medium acidity compared to zeolites, and their morphological properties are tunable depending on the preparation method.^22^ The origin of the Brønsted acidity in ASA is still debated. The two most prominent views are that acidity is (i) due to a few strong Brønsted acid sites (BAS), likely due to Al inclusion in the silica network, or (ii) due to many weaker sites induced by perturbation of silanol groups by strong Lewis acid Al^3+^ surface sites.^24,25^ Among the many synthesis methods available, homogeneous deposition-precipitation (HDP) is an approach that involves mainly the grafting of hydrolyzed Al-aqua complexes on silanol groups of the surface of the SiO_2_ support.^26,27^

**Figure 1:**
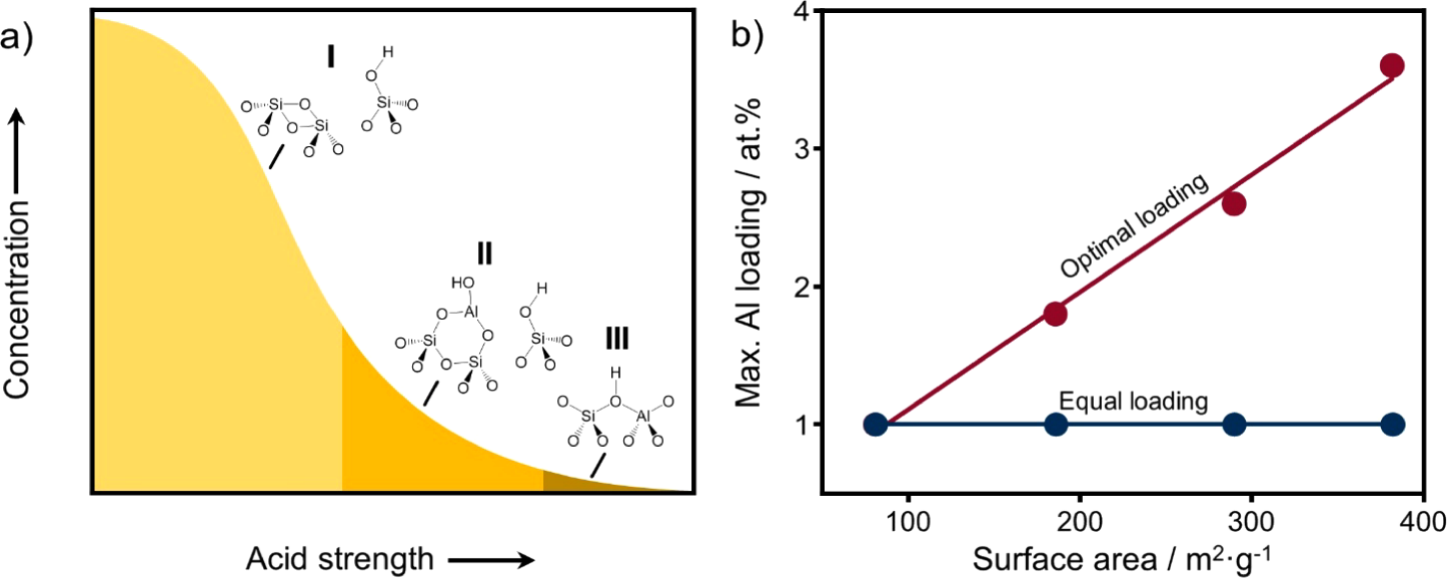
Approach followed in this study: (a) three types of BAS found in ASA (I—nearly nonacidic silanol groups, II—silanol groups perturbed by Lewis acidic Al sites, III—bridging hydroxyl groups),^28^ (b) targeted Al loading of prepared ASA as a function of the silica surface area: optimal Al loading means a SiOH/Al ratio of 3. Equal loading refers to a constant Si/Al ratio of 103.

This work investigates ASA prepared via HDP by grafting different amounts of Al on fumed silica supports with varying surface areas, for which the amount of Al to be grafted was matched to the silanol density (Figure 1). We explored the impact of the surface area of the parent silica by using various fumed silica nanoparticles with surface areas between 90 and 380 m^2^·g^–1^. MAS NMR techniques were used to investigate structural changes in the materials after synthesis and calcination. The BAS were characterized by IR spectroscopy with CO as the probe molecule, and ^1^H and ^29^Si MAS NMR experiments were conducted to understand the nature of the BAS better. Finally, preliminary pyrolysis experiments using a thermogravimetric analysis apparatus were carried out to gain insights into potential relationships between the ASA properties and the catalytic cracking activity of ultrahigh molecular weight polyethylene (UHMw-PE). In separate pyrolysis GC-MS measurements, the impact of the catalyst on the product distribution was investigated. The work presented herein is also available as part of a PhD dissertation.^29^

## Results and Discussion

ASA was synthesized using the HDP procedure at relatively low Al loading.^24,30^ Poduval e*t al.* demonstrated that ASA contains three types of Brønsted acid sites (Figure 1): (I) silanols of weak acidic strength, (II) silanol groups activated by nearby Lewis acidic Al sites of weak-to-medium strength, and (III) bridged OH species of zeolitic strength. The latter are most likely associated with Al diffusing into the silica network brought about in the calcination step (Figure 1a).^28^ In this work, a first set of samples was prepared aiming at a SiOH/Al ratio of 3 (optimal loading) to obtain ASA with the theoretical maximum amount of type II BAS (Figure 1b). Another set of ASA samples was prepared with a Si/Al ratio of 103 (equal loading) while varying the surface area to investigate the effect of the distance between grafted Al. TGA was used to determine the silanol density of the parent fumed silicas, which ranged from 0.5 mmol·g^–1^ for SiO_2_-90 to 1.8 mmol·g^–1^ for SiO_2_-380.

The Al loadings of the prepared ASA samples are listed in Table 1. We determined the Al loading of the as-prepared samples before calcination and assumed that calcination did not affect the Al loading. The measured Si/Al ratios correspond well with the targeted values. For the samples prepared at a constant Si/Al ≈ 103, the amount of grafted Al increases slightly with the surface area of the parent SiO_2_. The N_2_ physisorption experiments were conducted to investigate the surface area and pore volumes, and the isotherms are given in Figure S1. The surface areas of the calcined ASA samples decrease with increasing Al loading and temperature. The total pore volumes slightly increase upon grafting of Al and are hardly affected by the calcination temperature (Table 1). Additional N_2_ and Ar physisorption isotherms measurements for SiO_2_-300 and ASA-300–103–500 on another physisorption apparatus were used to confirm the unexpected result of a higher pore volume of the Al-loaded samples (Table S1). The increase in pore volume upon grafting can also be appreciated from the pore size distributions (Figure S2). It can be observed that the HDP grafting process led to some minor restructuring of the SiO_2_ surface.

**Table 1:** Physicochemical Properties of the Parent SiO_2_ and Thereof Derived ASA Samples Using HDP and Calcination

Sample	*C*_Al_^a^	Si/Al^a^	*T*_calcination_	*S*_BET_^b^	*V*_total_^c^
	μmol·g^–1^		°C	m^2^·g^–1^	cm^3^·g^–1^
SiO_2_-90	-	-	-	76	0.1
ASA-90-103	136	122	-	-	-
	n.d.^d^	n.d.	500	77	0.3
	n.d.	n.d.	700	81	0.3
SiO_2_-200	-	-	-	174	0.3
ASA-200-103	150	111	-	-	-
	n.d.	n.d.	500	184	0.9
	n.d.	n.d.	700	165	1.0
ASA-200-55	269	61	-	-	-
	n.d.	n.d.	500	180	1.1
	n.d.	n.d.	700	178	0.8
SiO_2_-300	-	-	-	310	0.6
ASA-300-103	147	113	-	-	-
	n.d.	n.d.	500	300	0.9
	n.d.	n.d.	700	296	1.6
ASA-300-38	383	43	-	-	-
	n.d.	n.d.	500	266	1.2
	n.d.	n.d.	700	261	1.2
SiO_2_-380	-	-	-	382	0.7
ASA-380-103	154	108	-	-	-
	n.d.	n.d.	500	348	1.3
	n.d.	n.d.	700	323	1.3
ASA-380-27	546	30	-	-	-
	n.d.	n.d.	500	312	1.1
	n.d.	n.d.	700	278	1.0

a Determined by elemental analysis of uncalcined ASA samples.

b BET surface area.

c Total pore volume calculated at p/p_0_ = 0.98 based on N_2_ physisorption isotherms of calcined samples.

d Not determined.

TEM images show the typical morphology of fumed silica as small spherical particles, which decrease in size with increasing surface area and are in the order of 100 nm for SiO_2_-90 and 10 nm for SiO_2_-380 (Figure S3). Figure S4 shows representative TEM images of the ASA samples derived from SiO_2_-200. No significant structural changes are observed upon grafting different amounts of Al and subsequent calcination at 500 and 700 °C. The primary particle size of the various samples remains ∼20 nm, similar to the size of the parent SiO_2_. This was also confirmed by analyzing other ASA samples (Figure S5).

**Figure 2:**
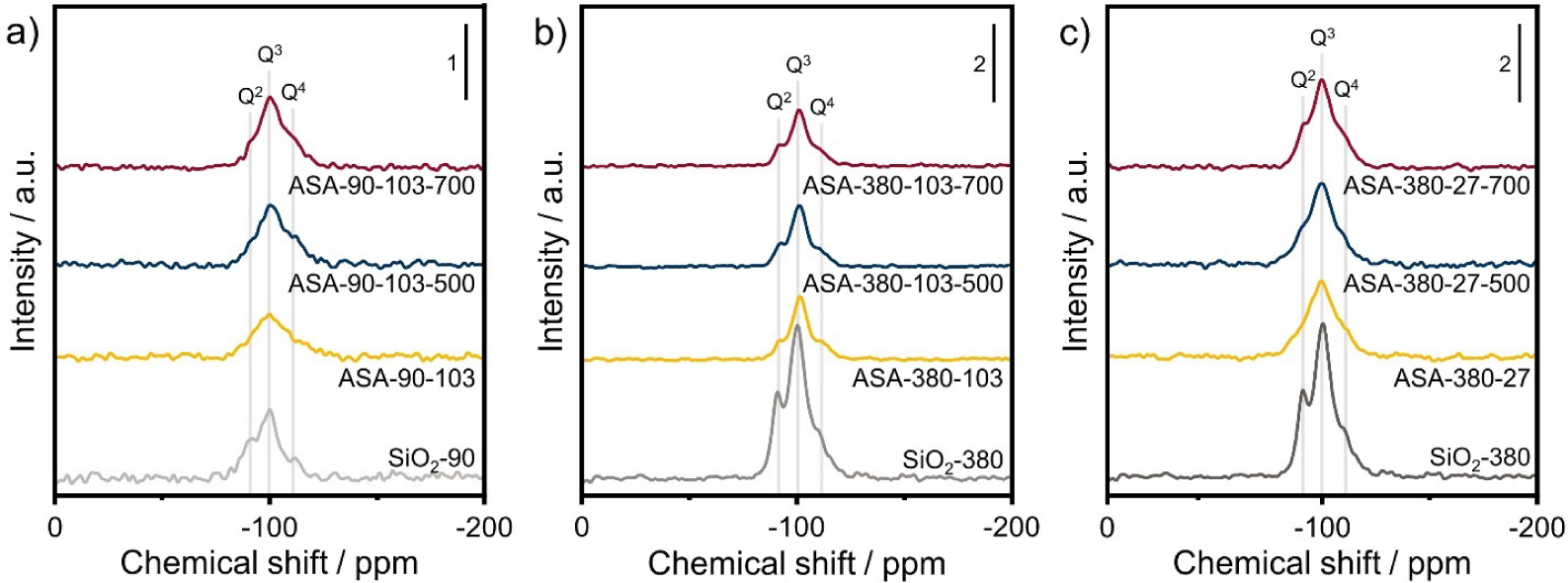
^29^Si CP MAS NMR spectra of (a) SiO_2_-90, (b) SiO_2_-380, and (c) SiO_2_-380 and the ASA derived thereof obtained by Al grafting after drying and calcination at 500 and 700 °C with (a and b) Si/Al ratios of 103 and (c) a ratio of 27.

^29^Si CP MAS NMR was used to investigate the surface Si species. This method makes use of dipolar interactions between ^1^H and ^29^Si. Figure S6 shows the NMR spectra of the SiO_2_ supports. Three distinct bands can be discerned due to Q^4^ (−110 ppm), Q^3^ (−100 ppm), and Q^2^ (90 ppm) Si^4+^. The intensity of these CP MAS NMR features increases with the surface area as the signals of silanol groups become more pronounced than those of bulk species. Figure 2 shows the ^29^Si CP MAS NMR spectra of the ASA samples obtained by Al grafting and calcination at 500 and 700 °C. Intensity differences between the various CP MAS spectra can only be discussed qualitatively. The parent SiO_2_ samples exhibit pronounced features due to the silanol groups. The decrease in the Q^2^/Q^3^ ratio upon grafting indicates the preferential adsorption of Al on geminal silanols (Q^2^), which is further impacted by the Al content, as can be judged by comparing the ASA-380–103 and ASA-380–27 samples (Figure 2b,c). The slight variations in the Si coordination upon calcination indicate minor changes in the Si and Al coordination.

**Figure 3:**
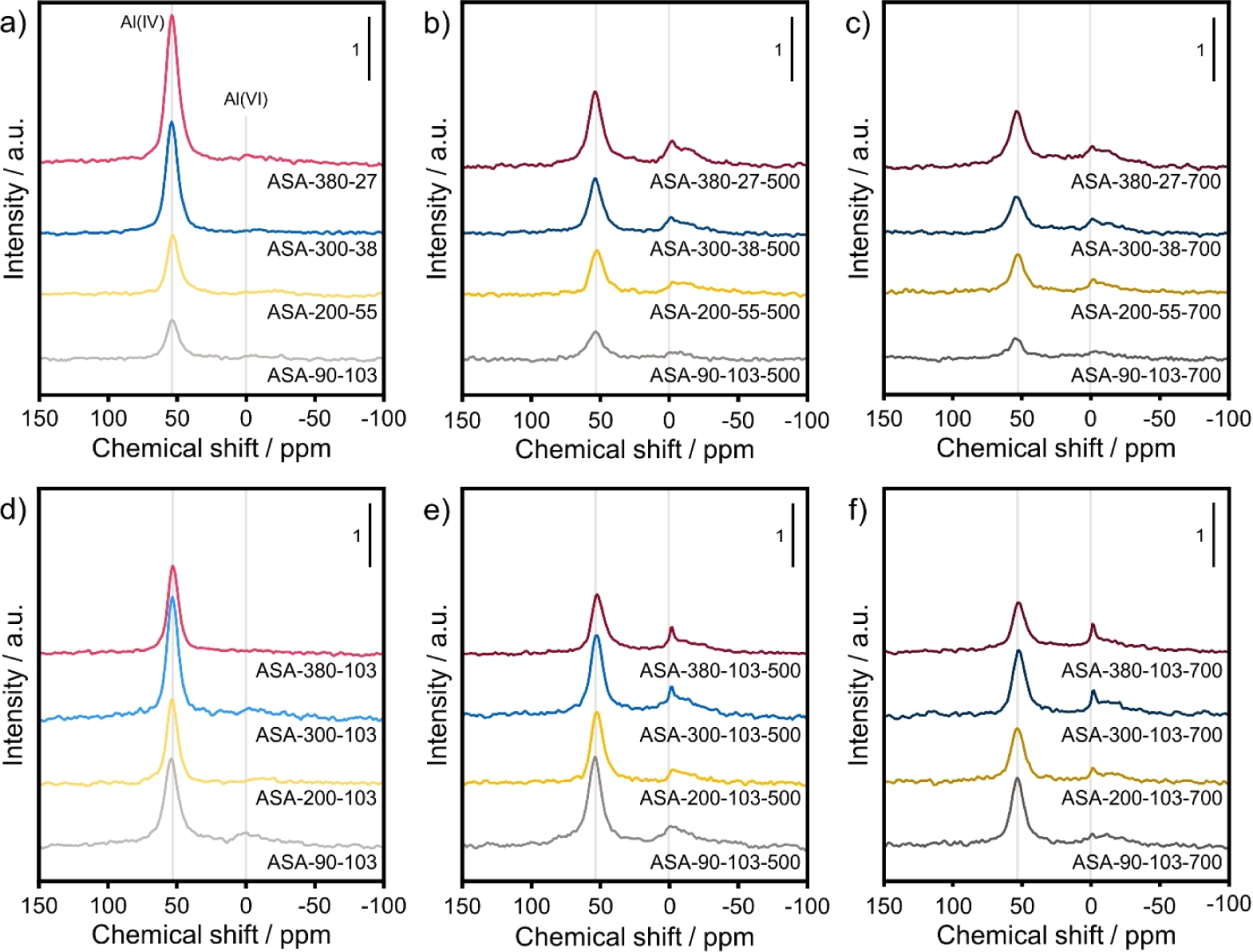
^27^Al MAS NMR spectra of (a) as-prepared ASA with optimal Al loading and after calcination at (b) 500 °C, and (c) 700 °C (a–c: 4096 scans), (d) as-prepared ASA with equal Al loading and after calcination at (e) 500 °C and (f) 700 °C (d–f: 8192 scans).

The Al coordination of the calcination ASA samples was studied by ^27^Al MAS NMR spectroscopy. First, the spectra of the as-prepared samples are discussed. Figure 3a shows that these spectra are dominated by a symmetric peak at 54 ppm due to tetrahedral Al(IV). The absence of a clear feature around 0 ppm due to octahedral Al(VI) for the as-prepared samples is in line with the literature.^24^ Upon calcination, part of the Al (IV) is converted to Al(VI), which can indicate the formation of Al–O–Al bonds (Figure 3b). The spectra for the samples with an equal Al loading are comparable with a small Al(VI) contribution around 0 ppm, which is relatively broad. A further decrease in the Al(IV) intensity is observed upon increasing the calcination temperature from 500 to 700 °C, whereas the relative contribution of Al (VI) is unchanged (Figure 3c). More scans were recorded for this set of samples to improve the resolution of the spectra of the ASA with equal Al loading. As expected, these spectra are mainly defined by an Al (IV) contribution (Figure 3d). However, the higher resolution shows that some Al (VI) may be present in ASA-90–103. In contrast to the optimal Al loading samples, considerable changes are observed for the calcined ASA with equal Al loading and increasing surface area (Figure 3e). Around 0 ppm, the Al (VI) peak becomes more defined with decreasing Al surface density. Figure 3f shows that increasing the calcination temperature from 500 to 700 °C does not affect the Al (VI) intensity. However, this contribution becomes sharper for the ASA with a low surface area. The overall Al(IV) intensity decreases with increasing calcination temperature for all 4 samples (Figure 3f). The observation that the Al(VI) peak does not significantly increase upon calcination at 700 °C indicates that the dispersed Al^3+^ species do not agglomerate into Al_2_O_3_ domains.

To further investigate the presence of Al_2_O_3_ domains in the calcined ASA samples, the ^27^Al MAS NMR spectra of some samples are compared to ^27^Al MAS NMR spectra obtained after dehydration and exposure to NH_3_.^31,32^ The spectra in Figure S7 clearly show that all the Al(VI) species in the calcined samples revert to Al(IV) coordination upon exposure to NH_3_. For comparison, the ^27^Al MAS NMR spectra of γ-Al_2_O_3_ before and after NH_3_ exposure are given in Figure S8, showing that most of the Al(VI) species do not change their coordination. As the samples in Figure S7 are representative for the whole sample set, it is reasonable to conclude that none of the ASA contain significant amounts of Al_2_O_3_.

A sample with a nearly double Al loading (Si/Al = 15) was prepared using SiO_2_-380 to verify whether, at Al loadings above the assumed theoretical amount for generating maximum acidity (SiOH/Al < 3), octahedral Al is formed. The ^27^Al MAS NMR spectrum of the dried sample already shows a clear Al(VI) feature, which increased upon calcination (Figure S9). This sample also shows a feature around 30 ppm upon calcination at 700 °C, which can be attributed to distorted Al(IV) or penta-coordinated Al(V). The latter species have been associated with the interface between Al_2_O_3_ domains and SiO_2_ in ASA prepared by HDP of Al on SiO_2_.^24^ The NH_3_ treatment of ASA-380-15-500 reveals that a fraction of Al retains its octahedral coordination, further demonstrating that Al_2_O_3_ domains are formed, when the Al content grafted is higher than the one corresponding to a silanol/Al ratio of 3 (Figure S9).

**Figure 4:**
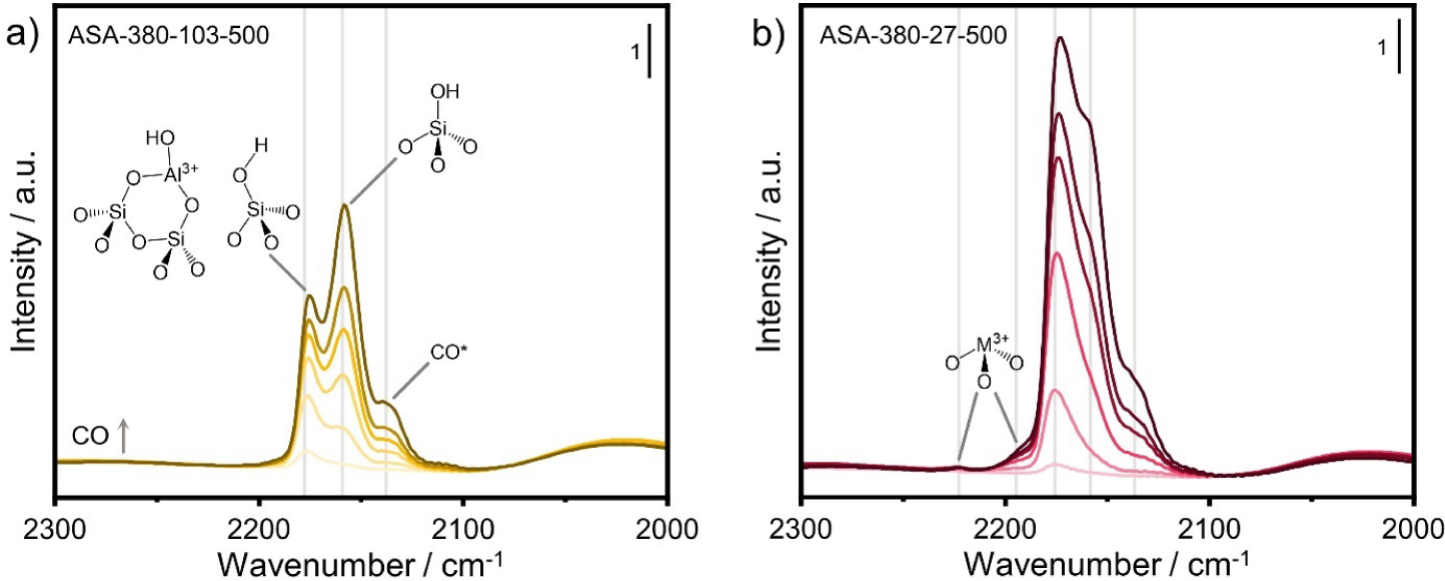
CO IR spectra of (a) ASA-380-103-500 and (b) ASA-380-27-500 with increasing CO partial pressure (IR spectra recorded at liquid N_2_ temperature).

In a previous study,^28^ different characterization techniques were compared to unravel the nature of the acid sites in ASA prepared by HDP. CO IR spectroscopy was used to determine the acidity of the ASA samples, because this method can differentiate between BAS and LAS. Figure 4 shows the corresponding IR spectra of ASA-380-103-500 and ASA-380-27-500. In both samples, a band develops at 2176 cm^–1^ at low CO coverage due to CO interacting with BAS.^28^ The intensity of this band increases with increasing CO coverage. The minor shifts in the location of this band reflect small variations in the strength of the BAS. For example, the band is located at 2175 cm^–1^ in ASA-380-103-500 and 2173 cm^–1^ in ASA-380-27-500. At higher CO coverage, additional bands appear at 2159 and 2137 cm^–1^ due to the interaction of CO with silanols and physisorbed CO, respectively (Figure 4). The strong silanol contribution for the ASA-380-103-500 sample with a lower Al loading can be related to the larger number of free silanol groups. ASA-380-27-500, on the other hand, contains weak bands at 2222 and 2191 cm^–1^ due to coordinatively unsaturated Al atoms, which are LAS. The IR spectra obtained saturation of the CO stretching region were deconvoluted to determine the BAS concentration using a molar extinction coefficient of 2.6 cm·μmol^–1^.^28^ The results are given in Table S2. The deconvolution procedure involved fitting the spectra obtained at low CO coverage, followed by fitting the spectra at higher CO coverage with the same fitting model until the BAS-related band no longer changed in intensity (Figure S10).

**Figure 5:**
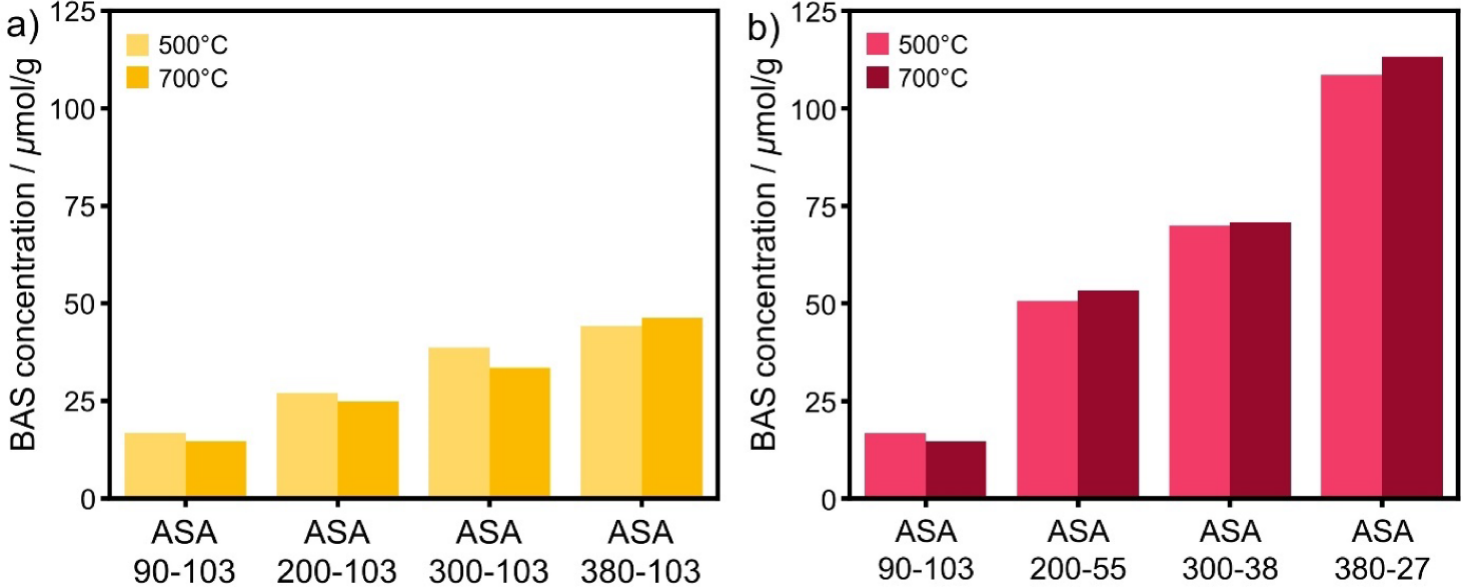
BAS concentration of ASA with (a) equal and (b) optimal Al loading calcined at 500 and 700 °C determined by deconvolution of CO-saturated spectra.

**Figure 6:**
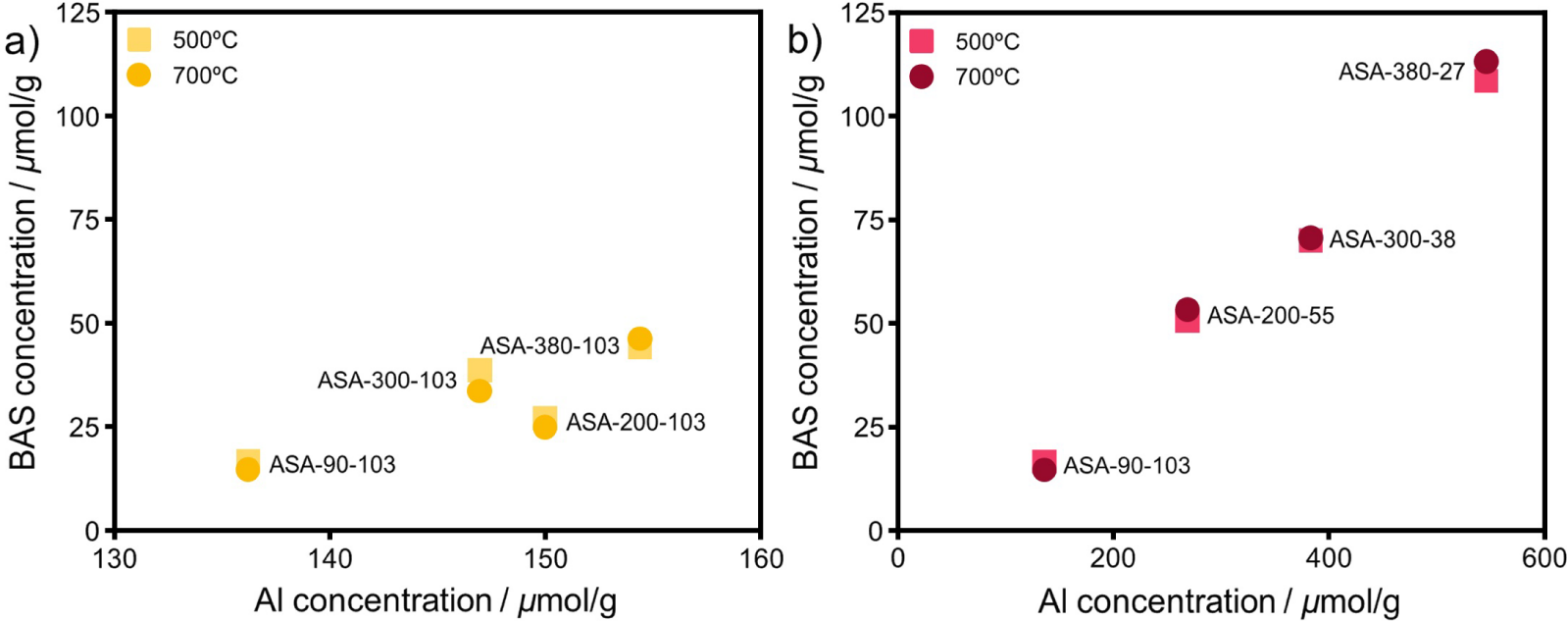
BAS concentration as a function of Al concentration of a) samples with an equal Al content and b) samples with optimized Al loading calcined at 500 and 700 °C.

Figure 5 shows the BAS concentration for the ASA samples calcined at 500 and 700 °C. The BAS concentration is hardly affected by the calcination temperature. Furthermore, we observe an increase in the BAS concentration with surface area for the samples with equal Al loading (Figure 5a). For example, ASA-90-103-700 contains 15 μmol·g^–1^ BAS, substantially lower than the value of 46 μmol·g^–1^ in ASA-380-103-700. Figure 5b shows that the BAS concentration of ASA increases with Al loading. Increasing Al loading comparing ASA-380-103-500 and ASA-380-27-500 increases the BAS concentration from 44 to 108 μmol·g^–1^ (Table S2). Figure 6 shows the BAS concentration as a function of Al loading for ASA with equal and optimal Al loading. Figure 6a shows how the surface area impacts BAS formation for the samples with equal Al loading. Figure 6b depicts a linear correlation between the BAS concentration and the Al loading for samples prepared at a SiOH/Al ratio of 3 (optimal loading). Despite this, not all grafted Al gave rise to BAS. When comparing the BAS/Al, ∼10–30% of the Al atoms in the ASA-*x*-103-*z* (equal loading) samples yield BAS (Table S2). For the samples with an optimal loading, this fraction is ∼20%, independent of the Al loading (except for the ASA-90-103-*z* samples).

The Brønsted acidity situation in ASA is more complex than in zeolites, where the concentration of BAS, due to the presence of uniform bridged hydroxyl groups, can be directly related to the tetrahedral Al framework content.^33^ The acidity in ASA arises from a small amount of zeolite-like strong acid sites and a relatively large amount of weaker sites due to the perturbation of weakly acidic SiOH groups by strong Lewis acid sites (i.e., Al^3+^). It has been well established that not all tetrahedral Al give rise to BAS in ASA.^28^ This is also evident for the set of ASA in this study. The low BAS/Al ratio cannot be explained by clustering Al into Al_2_O_3_ domains, as they are absent according to^27^Al MAS NMR (Figure S7). Another possible explanation is that the local coordination environment of some tetrahedral Al at the SiO_2_ surface does not allow for suitable interactions with nearby silanol groups to form the Al-HO-Si pairs needed to generate BAS. This is probably due to the ill-defined nature of the amorphous SiO_2_ surface. Moreover, it has been noted that there might also be significant differences in Al loading on different particles for this type of ASA, which can be due to mesoscale heterogeneities of fumed SiO_2_.^24^ Although we did not focus on the small fraction of strong BAS, the slight shifts in the CO IR band with CO coverage (Table S2) suggest that such sites may be present in our samples.^28^

To monitor the evolution of BAS in these ASA samples, we subjected as-synthesized ASA-380-27 to calcination in O_2_/He in an IR cell at increasing temperature, while intermittently using CO IR analysis at liquid N_2_ temperature to study the surface composition (Figure S11). After dehydration of as-synthesized ASA-380-27 at 150 °C for 1 h, the IR spectrum only contains bands related to CO interacting with silanols (∼2158 cm^–1^) and physisorbed CO (∼2127 cm^–1^). Inspection of the hydroxyl region shows the silanol feature at ∼3747 cm^–1^ and a broad band due to associated water at 3670 cm^–1^. Weak bands in this region can be related to different surface species interacting with physisorbed water or surface hydroxyls, including urea and ammonia introduced during HDP preparation. Only minor changes are observed in the spectra obtained after calcination at 200 °C. Upon increasing the temperature to 300 °C, a feature at ∼2172 cm^–1^ due to stronger BAS appears. Nevertheless, the silanol band remains dominant in the IR spectrum. Calcination at 400 °C leads to substantial changes in the IR spectrum. The feature at 2172 cm^–1^ becomes more intense, together with the appearance of a feature at 2225 cm^–1,^ due to CO coordinating to coordinatively unsaturated Al^3+^. Notably, this IR spectrum is significantly different from the IR spectrum of the ASA-380-27-500 sample, obtained by calcination at 500 °C (Figure 5). A comparison of the IR spectra as a function of the CO coverage shows that the CO band due to BAS appears initially at 2172 cm^–1^ for the in situ calcination experiment, while it starts to appear at 2175 cm^–1^ for ASA-380-27-500. The OH stretch region for ASA-380-27-500 during CO dosing (Figure S12) is very similar to that of the *in situ* calcined ASA-380-27 sample. Thus, it is reasonable to conclude that removing H_2_O during calcination frees up Lewis acidic Al sites, which can interact with the silanol groups, thereby giving rise to BAS. This experiment confirms that calcination is critical in forming BAS, strongly suggesting that the medium-strength BAS arise from removing H_2_O molecules from the grafted Al species, which increases their Lewis acidity.

These IR findings clearly show that calcination is required to form BAS. To study these acid sites in more detail in the context of Figure 1, ^1^H Hahn-echo MAS NMR spectra were measured. Figure S13 shows the NMR spectra of SiO_2_-380 and ASA-380-27-500 after evacuation at 400 °C, which reveals a dominant silanol feature at 1.6 ppm for both samples. Compared to SiO_2_-380, the spectrum of ASA-380-27-500 contains additional peaks related to aluminols (2.4 ppm) and BAS (3.6 ppm).^28,34,35^ The small number of aluminol (Al–OH) groups might be associated with some clustered Al–O–Al species. On the other hand, dissociative water adsorption on coordinatively unsaturated Al sites can also lead to such sites. Indeed, the band around 6.4 ppm in the ^1^H MAS NMR spectra shows that water still binds to BAS or LAS.^36−38^ We use the downfield shift of these adsorbed water molecules to study the BAS by using cross-polarization MAS NMR. For this purpose, hydrated samples were pretreated at 150 °C for 1 h under vacuum to remove physisorbed water. Figure S13 shows a broad shoulder of physisorbed water at 3.1 ppm next to the silanol peak at 1.6 ppm.^39^ The most apparent difference between the spectra for SiO_2_-380 and ASA-380-27-500 is the presence of a feature at 6.4 ppm in the ASA spectrum, which can be assigned to water interacting with LAS or BAS.^37,38^

^1^H–^27^Al TRAPDOR is an NMR technique that can decouple protons from neighboring Al atoms by comparing the intensities with and without ^27^Al irradiation.^40^ The more significant the difference in intensity between the obtained spectra, the closer the distance between the nuclei. Figure S14 compares ^1^H NMR spectra with and without ^27^Al irradiation. The change in ^1^H intensity of the bands at 3.6 and 6.4 ppm upon^27^Al irradiation shows that these protons are near Al atoms. Next, 2D MAS NMR experiments were carried out to investigate the interaction between different nuclei. 2D ^1^H–^27^Al CP MAS NMR proved unsuccessful due to poor cross-polarization, resulting in low signal-to-noise ratios. Figure S15 shows 2D ^1^H–^29^Si CP MAS NMR spectra of SiO_2_-380 and ASA-380-27-500. The band associated with the BAS-H_2_O complex at 6.4 ppm in the F1 dimension in the 2D spectrum of ASA-380-27-500 correlates with Q^3^ silicon at 101 ppm in the F2 dimension. This signal is absent in the corresponding spectrum for SiO_2_-380. This difference is consistent with the proposed model of BAS in Figure 1, although it does not allow us to distinguish between BAS of type I or II. The cross-polarizability was too weak when these measurements were repeated for dehydrated samples, which is related to the lower H_2_O content.

The ^1^H–^27^Al TRAPDOR spectra (Figure S14) confirm that the ^1^H band at 6.4 ppm is related to Al, while the 2D ^1^H–^29^Si CP MAS NMR spectrum (Figure S15) reveals the correlation with Q^3^ Si. To study the interaction between the various protons on the ASA surface, a 2D ^1^H–^1^H CP MAS NMR spectrum was recorded. The 1D projections in Figure S16 correspond to the 1D Hahn-echo spectra in Figure S13, which are represented by the intensities on the diagonal. The off-diagonal interactions show that the BAS-H_2_O peak at 6.4 ppm correlates with the silanols at 1.6 ppm. The broad tails indicated by the arrows in Figure S16 suggest that these protonic species are related to BAS and aluminols. Although this means that we cannot exclude that the 6.4 ppm signal can also be due to interactions with AlOH species, the correlation between BAS sites due to an interaction of silanols with Al is evident.

**Figure 7:**
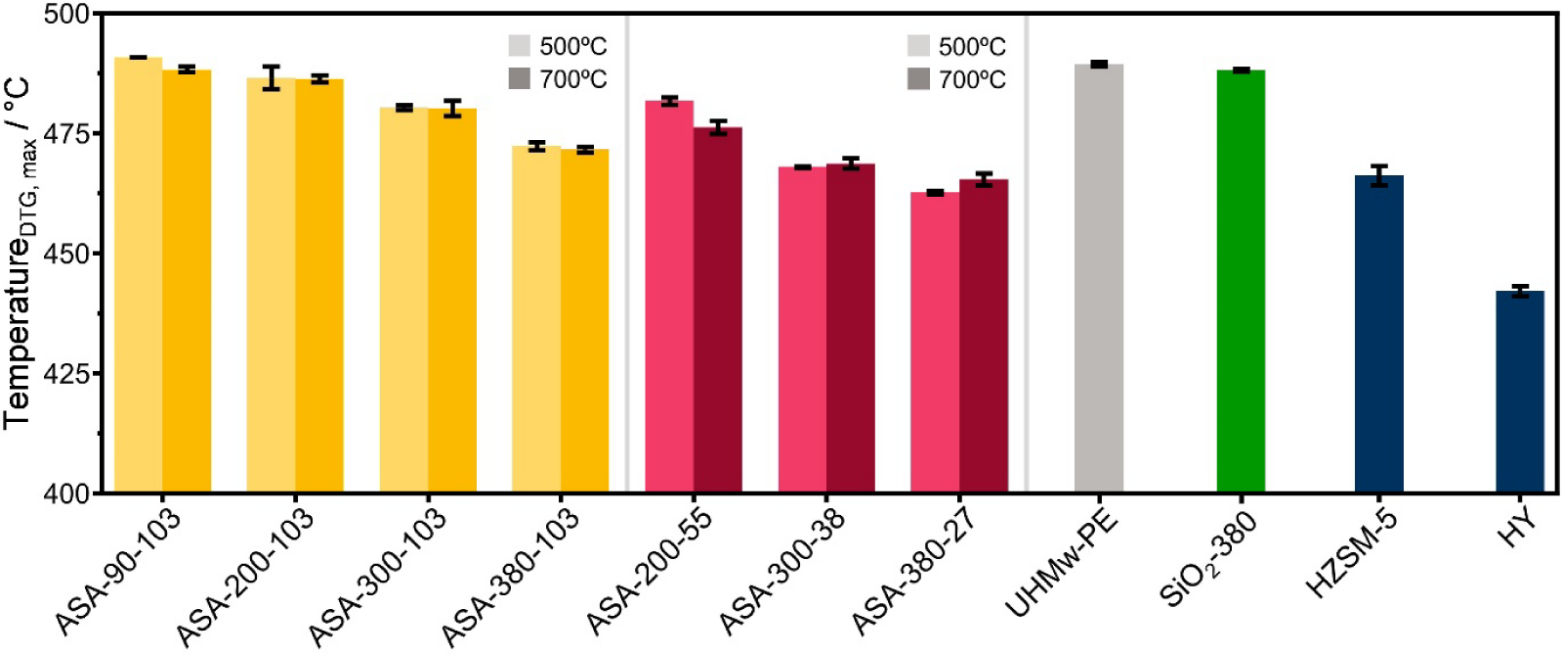
DTG maximum during TGA of UHMw-PE of the catalysts investigated by TGA (conditions: 5 mg polymer, 0.5 mg catalyst, 150 mL·min^–1^ He, temperature 50–800 °C, rate 20 °C·min^–1^).

The performance of the ASA samples as acid catalysts was then gauged by carrying out pyrolytic cracking of ultrahigh molecular weight polyethylene (UHMw-PE, *M*_w_ = 3–6 × 10^6^ g·mol^–1^). These experiments were conducted in a TGA under a He atmosphere by heating 5 mg polymer-catalyst mixtures (4.5 mg UHMw-PE, 0.5 mg catalyst, *T* = 50–800 °C, 20 °C·min^–1^). The TGA curves are given in Figure S17. We use the maximum in the differential thermogravimetric (DTG) curve, i.e., the temperature at which the weight loss rate is the highest (*T*_DTG, max_), to estimate the activity. This method is commonly used to reflect the changes in pyrolysis rates as a function of variations in the feed composition and the impact of catalysts, including variations in their acidity.^41−44^ This approach provides a crude estimate of the catalytic performance in plastics cracking. MFI zeolite (HZSM-5, Si/Al = 13) and faujasite zeolite (HY, Si/Al = 2.4) were reference catalysts. Figure 7 shows the results of the TGA-pyrolysis experiments. A blank experiment with 5 mg polymer shows a *T*_DTG, max_ of UHMw-PE of ∼490 °C. The addition of SiO_2_-380 did not change the maximum in the DTG curve. Comparing the influence of ASA with equal Al loading shows a relatively small effect on the *T*_DTG, max_. The ASA-90–103 samples hardly affect the DTG curve compared to the blank (∼488–490 °C), while the ASA-380–103 samples result in a lower *T*_DTG, max_ of 472 °C. The calcination temperature of these samples does not influence the *T*_DTG, max_, which agrees well with the finding that the Brønsted acidity of these samples was unaffected by the calcination temperature. The samples with optimal Al loading are more active for UHMw-PE pyrolysis. The *T*_DTG, max_ decreases with increasing Al content. The ASA-380–27 samples with a *T*_DTG, max_ of ∼464 °C, are the most active ASA samples, which their highest BAS concentration can explain. HZSM-5 (pore size of 5.3 × 5.6 Å) led to a *T*_DTG, max_ of ∼466 °C, comparable to the optimal ASA sample. Nevertheless, HY with its large 7.4 Å windows and 13 Å supercages exhibited the highest pyrolysis activity (*T*_DTG, max_ = ∼442 °C). We note that the overall acidity of the HY sample is low compared to steam-activated HY zeolites, given its high framework Al density and the absence of mesopores.

**Figure 8:**
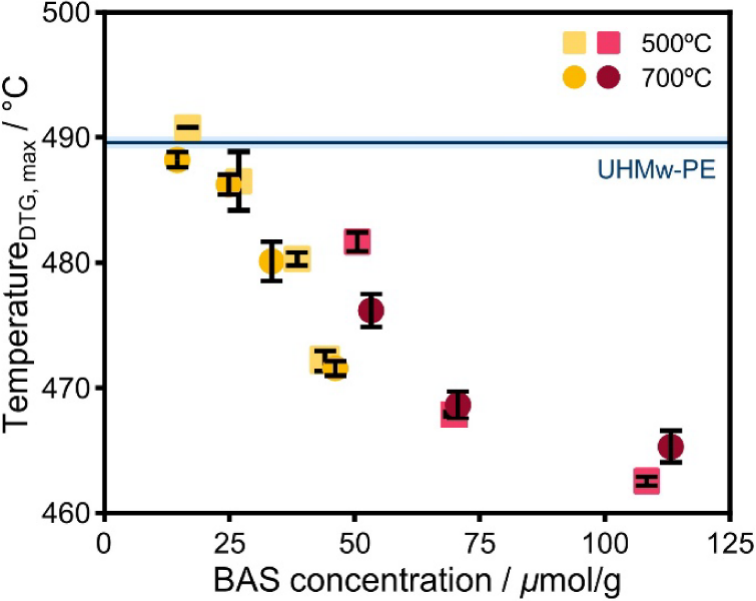
T_*DTG, max*_ as a function of the BAS concentration with equal (yellow) and optimal (red) Al loading and calcined at 500 and 700 °C.

Figure 8 shows a near-linear decrease of T_DTG, max_ with increasing BAS concentration for both sets of ASA samples. The *T*_DTG, max_ for the ASA samples with optimal loading is approximately 10 °C lower than for the ASA with equal Al loading. Besides the dominant influence of the BAS concentration, the surface area also significantly impacts the pyrolysis activity. The ASA-380–103 samples achieve a nearly similar decrease in the pyrolysis temperature as the ASA-300-38 ones, despite the latter samples having roughly twice the amount of BAS. It is reasonable to attribute the higher activity to the improved adsorption of the long polymer chains, which enhances the cracking on acid sites. Moreover, the effect of pore accessibility is evident from a comparison between the mesoporous ASA and microporous zeolite references. The medium-pore HZSM-5 zeolite achieved a similar *T*_DTG, max_ as the ASA. In contrast, the large-pore HY zeolite was significantly more active, although its acidity is probably lower due to the low Si/Al ratio. Diffusion of the highly viscous (i.e., high molecular weight) polymer melt into molecular-sized pores often limits the cracking rate.^12,16^ As a result, optimized ASA with the BAS exclusively in mesopores are a promising alternative to commonly used zeolites in the pyrolysis of plastic waste.

To gain insight into differences in the product distribution during pyrolysis of UHMw-PE by ASA and zeolites, pyrolysis GC-MS experiments were conducted at 450, 500, and 550 °C. The blank measurements of UHMw-PE (no catalyst) show that the chain length of the hydrocarbons decreases with increasing pyrolysis temperature (Figure S18). While mainly C_10_–C_31_ hydrocarbons are obtained at 450 °C, the main products at 550 °C are C_1_–C_9_ hydrocarbons. The addition of SiO_2_-380 did not markedly change the product distribution, which aligns with the negligible impact of the parent SiO_2_ on the *T*_DTG, max_. This also holds for ASA-90-103-500 for pyrolysis temperatures of 450 and 500 °C. More C_1_–C_7_ hydrocarbons were obtained for this sample at 550 °C compared to the SiO_2_-380 sample. ASA-380-103-500 and ASA-380-27-500 gave a higher yield of small hydrocarbons at 500 °C. The pyrolysis GC-MS measurements show the better performance of HZSM-5 and HY as cracking catalysts. At 450 °C, both catalysts mainly yield C_1_–C_7_ hydrocarbons and aromatic compounds, which were not observed with the ASA catalysts. The large-pore structure of HY is reflected in a higher aromatic yield compared to the case with HZSM-5.

Analysis of the coke content of the samples after the pyrolysis GC-MS experiments shows significant differences between ASA and zeolite samples. The higher propensity to coking on the more acidic zeolites is evident from the black color compared to the off-white color of the used ASA samples, indicative of lesser retention of heavy products. The spent catalyst samples were subjected to TGA in air (Figure S19). ASA lacked substantial combustion features of heavy hydrocarbons. The gradual weight loss over the whole temperature range is likely due to surface dehydroxylation.^45^ The TGA curves of HZSM-5 and HY contain weight loss features due to coke combustion at temperatures between 495 and 535 °C. The coke content of the HY zeolite (13%) is substantially higher than that of the HZSM-5 zeolite (3%). The larger amount of coke on the zeolites results from their higher acidity compared to ASA. The medium-pore zeolites of HZSM-5 suppress coke formation compared to the large pores of HY. Specifically, the formation of polyaromatics can cause the deactivation of the zeolite catalysts.^37,38^ ASA’s comparable cracking activity, combined with a lower coking propensity and a higher yield of liquid hydrocarbons than the zeolites, highlights its potential for UHMw-PE pyrolysis.

## Conclusion

In this work, we carefully designed two sets of ASA samples based on Al grafting on SiO_2_ nanoparticles with surface areas in the range of 90–380 m^2^·g^–1^ with either an optimal Al loading aimed at obtaining a high BAS density or with equal Al loading to study the effect of the distance between BAS and the SiO_2_ nanoparticle size. The grafted Al was predominantly tetrahedral upon drying. Calcination resulted in the appearance of octahedrally coordinated Al. ^27^Al NMR measurements after NH_3_ adsorption showed that nearly all Al atoms were isolated, i.e., the number of Al_2_O_3_ domains due to agglomeration of Al during calcination was negligible. Only when considerably more Al was grafted, such Al_2_O_3_ domains were observed. CO IR spectroscopy showed the presence of BAS next to silanols and a small number of coordinatively unsaturated Al sites. Overall, the ASA with optimal Al loading contained more BAS. For both sets, the ratio of BAS generated per Al was between 0.1 and 0.3, independent of the calcination temperature, even though these samples were prepared in a well-defined manner. This indicates the limitations in forming BAS due to interactions between Lewis acidic Al and silanol groups on the ill-defined amorphous SiO_2_ surface. The ASA samples showed promising performance in the pyrolysis of UHMw-PE. The pyrolysis temperature decreased with increasing Brønsted acidity of the samples, while the samples with a higher surface area were also more active, presumably due to stronger adsorption of the polymer chains. The best ASA samples exhibited a performance comparable to HZSM-5, albeit the activity of HY was substantially better. In contrast to zeolites, which produce hydrocarbons and aromatic compounds, ASA mainly yields longer hydrocarbons. With a lower tendency to coke deposition, ASA appears to be a promising alternative to zeolite for the pyrolysis of polyolefins.

## Experimental Section

### Preparation of Materials

Fumed SiO_2_ (Aerosil) with surface areas ranging from 90 and 380 m^2^ g^–1^ were generously supplied by Evonik. The silanol density of the silicas was calculated from the weight loss observed between 200 and 1000 °C using thermogravimetric analysis. In a typical experiment, a 20 mg sample was heated to 1000 °C at 5 °C/min in a mixture of 33 vol % O_2_ in He.

Amorphous silica–alumina (ASA) materials were synthesized following a homogeneous deposition procedure.^24,30^ Typically, 30 g of commercial silica was suspended in 1 L aqueous urea solution (0.76 M). A desired amount of Al(NO3)3·9H2O (Merck, purity 99%) was added to the resulting suspension. The mixture was heated to 90 °C in a stirred double-walled reaction vessel while continuously monitoring the pH. Once the pH reached 6, the mixture was removed from the reactor and cooled in an ice bath. Finally, the ASA was separated through filtration and washed with ultrapure water. Most of the water was evaporated during overnight static drying at 110 °C. Portions of the solids were then calcined at either 500 or 700 °C (0.5 °C·min^–1^) for 5 h in static air. The samples were labeled as ASA-*X*-*Y*-*Z*, with *X* being the surface area of the silica, *Y* the Si/Al ratio, and *Z* the calcination temperature. HZSM-5 (Si/Al ratio = 13, Süd-Chemie, *C*_BAS_ = 950 μmol·g^–1^; *C*_LAS_ = 107 μmol·g^–1^, *S*_BET_ = 321 m^2^·g^–1^; *V*_micropore_ = 0.11 cm^3^·g^–1^),^46^ HY (Si/Al ratio = 2.4, Zeolyst, *C*_BAS_ = 5,600 μmol·g^–1^, *S*_BET_ = 573 m^2^·g^–1^, *V*_micropore_ = 0.22 cm^3^·g^–1^, *V*_mesopore_ = 0.06 cm^3^·g^–1^),^47^ and γ-Al_2_O_3_ (CK-300, Akzo Nobel, *S*_BET_ = 267 m^2^·g^–1^)^46^ served as reference end members to these ASAs. The zeolites were obtained by calcinating their as-received NH_4_ form. The standard calcination procedure was as follows: heating in static air from room temperature to 500 °C (0.5 °C·min^–1^) followed by a dwell of 5 h.

### Characterization

The metal loadings were assessed using ICP-OES analysis with a Spectro CIROS CCD ICP optical emission spectrometer featuring axial plasma viewing. Samples were dissolved in a 1:1:1 volumetric mixture of 40% HF in water, 60% HNO_3_ in water, and H_2_O.

N_2_ physisorption at −196 °C was employed to measure the surface area and pore volume using a Micromeritics TriStar II instrument. Before the measurements, samples were degassed at 300 °C for 8 h under nitrogen flow. Additional physisorption experiments with N_2_ at −196 °C and Ar at −189 °C were conducted using a Micromeritics ASAP 2020 apparatus, with samples pretreated for 8 h at 300 °C under vacuum. The specific surface area was determined using the BET method, while the mesopore volume was calculated from the adsorption branch of the isotherm using the Barrett–Joyner–Halenda (BJH) method.

Transmission electron microscopy (TEM) was conducted using a cryo-Titan (FEI/TFS) equipped with a Gatan imaging filter and Gatan camera operating at an acceleration voltage of 300 kV. The sample was dispersed in ethanol via ultrasonication before being placed on a holey Cu grid.

CO adsorption measurements were carried out on a Bruker Vertex 70v spectrometer with a resolution of 1 cm^–1^. Sample wafers were dehydrated at 400 °C in a flow of 33 vol % O_2_ in He for 1 h, and subsequently cooled to −183 °C. CO was introduced into the cell through a sample loop (50 μL) connected to a 6-way valve in small doses. An extinction coefficient of 2.6 cm·μmol^–1^ for adsorbed CO was used to quantify the amount of Brønsted acid sites (BAS).^28^

The aluminum speciation was examined using ^27^Al magic-angle spinning nuclear magnetic resonance (MAS NMR) spectroscopy. Measurements were conducted on an 11.7 T Bruker NEO500 NMR spectrometer with a 2.5 mm MAS probe spinning at 25 kHz. Spectra were acquired using a single pulse sequence featuring an 18° pulse with a duration of 1 μs and an interscan delay of 0.5 s. Before the experiments, the samples were hydrated in a desiccator.

^1^H NMR measurements were performed using a 4 mm MAS probe with a sample rotation rate of 10 kHz. The spectra were acquired using a Hahn-echo pulse sequence (p1-τ1-p2-τ2-aq) featuring a 90° pulse (p1 = 3.150 μs) and a 180° pulse (p2 = 2.5 μs). An interscan delay of 120 s was selected for quantitative spectra. Rotors for the high-temperature-treated samples were prepared in a glovebox.

^27^Al–^1^H Transfer of Population in Double Resonance (TRAPDOR) spectra were recorded with irradiation applied to the ^27^Al nuclei for 795 μs before the echo pulse, along with an interscan delay of 10 s. Samples were dehydrated under vacuum at 150 °C for 1 h or in a diluted O_2_ atmosphere (33 vol % in He) at 400 °C for 2 h.

MQMAS experiments utilized a three-pulse sequence (p1-t1-p2-τ-p3-t2) for triple-quantum generation and zero-quantum filtering. Strong pulses included p1 = 3.4 μs and p2 = 1.4 μs at a nutation frequency (ν1) of 100 kHz, while a soft pulse (p3 = 11 μs) was applied at ν1 = 8 kHz. The filter time (τ) was set to 20 μs, and the interscan delay was 0.2 s.

### Catalytic Pyrolysis of UHMw-PE

Pyrolysis of polyethylene was performed using a Mettler Toledo TGA/DSC 1 instrument. Approximately 5 mg of the sample, consisting of 90 wt % ultrahigh molecular weight polyethylene (Sigma-Aldrich, Mw = 3–6 × 10^6^ g·mol^–1^) and 10 wt % catalyst, was placed in an aluminum crucible. The polymer mixtures were melted by heating the crucible to 150 °C on a heating plate in air. For thermogravimetric analysis, a helium flow rate of 100 mL·min^–1^ was applied, with an additional 50 mL·min^–1^ of helium used as a protective gas. In the initial step of the measurements, the samples were heated to 50 °C and purged for 1 h to remove residual oxygen. Subsequently, the temperature was increased to 800 °C at 20 °C·min^–1^.

Pyrolysis GC-MS experiments were conducted on a Shimadzu GCMS-QP2020 NX equipped with a pyrolizer injector (Frontier Lab EGA/PY-3030) and autosampler (AS-2020E). The GC was outfitted with a capillary Rxi-5MS column (30 m length, 0.25 mm internal diameter, and 0.25 μm film thickness). H_2_ was used as carrier gas at a flow rate of 7.73 mL min^–1^ and the split ratio was 40. The GC run was performed using a temperature ramp starting at 40 °C (2 min isothermal) to 320 °C (2 min isothermal) at a heating rate of 40 °C·min^–1^. Between 0.5 and 1.0 mg of sample (polymer or polymer with 10 wt % catalyst) was pyrolyzed at three different temperatures (450, 500, and 550 °C). Samples were first subjected to pretreatment at 200 °C for 0.5 min (50 °C·min^–1^), and the temperature of the pyrolysis unit was subsequently raised to the desired set point before injecting the sample followed by a dwell of 1 min. The MS ion source and interface were set at 250 and 300 °C, respectively. The peaks in the obtained MS spectra (29 to 400 amu) were identified using the NIST library.

The amount of carbonaceous deposits on the catalyst following pyrolysis was analyzed using a Mettler Toledo TGA/DSC 1 instrument. In a typical analysis, 20 mg of the sample was heated to 1000 °C at a rate of 5 °C·min^–1^ in a flow of 33 vol % O_2_ in He. To ensure sufficient material for these experiments, 100 mg of the polymer-catalyst mixture was pyrolyzed in a tubular oven. The oven was purged at 50 °C for 1 h with a helium flow of 150 mL·min^–1^, after which the temperature was increased to 800 °C at a rate of 20 °C·min^–1^. The amount of carbon deposits was determined from the water-free amount of the sample.

## Supporting Information Available

Additional characterization data include N_2_ isotherms, pore size distributions, TEM images, solid-state NMR spectra, IR spectra, and results of N_2_ and Ar physisorption, CO IR peak deconvolution, TGA experiments, and pyrolysis GC-MS experiments (PDF)

## Abbreviations

ASA: amorphous silica–alumina
BAS: Brønsted acid sites
LAS: Lewis acid sites
MAS NMR: magic angle spinning nuclear magnetic resonance
CP: cross-polarization
MQ: multiquantum
TGA: thermogravimetric analysis
UHMw-PE: ultrahigh molecular weight polyethylene
DTG: differential thermogravimetric analysis
