# Supplementary material for: Isolating Al Surface Sites in Amorphous Silica–Alumina by Homogeneous Deposition of Al3+ on SiO2 Nanoparticles
Source: ACS Appl Nano Mater. 2024 Oct 31;7(22):25524–34. doi: 10.1021/acsanm.4c04544 (PMC11590055; doi:10.1021/acsanm.4c04544)
Supplement: Supplementary file 1 — an4c04544_si_001.pdf [file an4c04544_si_001.pdf]

Supporting Information

Isolating Al Surface Sites in Amorphous Silica-Alumina by Homogeneous Deposition of Al^{3+} on SiO_2 Nanoparticles

*Ferdy Coumans, Brahim Mezari, Norwin Zuidema, Jason M.J.J. Heinrichs, Emiel J.M. Hensen**

Laboratory of Inorganic Materials and Catalysis, Department of Chemical Engineering and Chemistry, Eindhoven University of Technology, PO Box 513, 5600 MB Eindhoven, The Netherlands

Corresponding Author

*E-mail: e.j.m.hensen@tue.nl

Table S1. Textural properties of ASA samples.

Sample	Nitrogen		Argon	
	$\boldsymbol{S}_{\mathbf{BET}}$[a] $m^2 \cdot g^{-1}$	$V_{total}{}^{b}$ $cm^3 \cdot g^{-1}$	$\boldsymbol{S}_{\mathbf{BET}}$[a] $m^2 \cdot g^{-1}$	$\boldsymbol{V}_{\mathbf{total}}{}^{b}$ $cm^3 \cdot g^{-1}$
SiO_2-300	287	0.6	229	0.5
ASA-300-38-500	-	-	187	1.1

[a] BET surface area, [b] Total pore volume calculated at p/p_0 = 0.98. SiO_2-300 was degassed under vacuum at 300˚C for 1 h in between N_2 and Ar physisorption measurements.

Table S2. Concentration of BAS and peak position of the activated CO-OH complex.

**Sample**	$N_{\mathbf{BAS}}$[a] μmol·g^{-1}	**BAS/Al**[b] **%**	V[c] *cm*$^{-1}$
ASA-90-103-500	17	12	2174
ASA-90-103-700	15	11	2174
ASA-200-103-500	27	18	2173
ASA-200-103-700	24	17	2172
ASA-200-55-500	51	19	2172
ASA-200-55-700	53	20	2169
ASA-300-103-500	39	26	2172
ASA-300-103-700	34	23	2173
ASA-300-38-500	70	18	2172
ASA-300-38-700	71	18	2173
ASA-380-103-500	44	29	2172
ASA-380-103-700	46	30	2171
ASA-380-27-500	108	20	2170
ASA-380-27-700	113	21	2169

[a] Concentration of BAS obtained after peak fitting of the CO saturated sample. [b] Fraction of BAS per Al atom. [c] IR band position of the activated CO-OH complex.

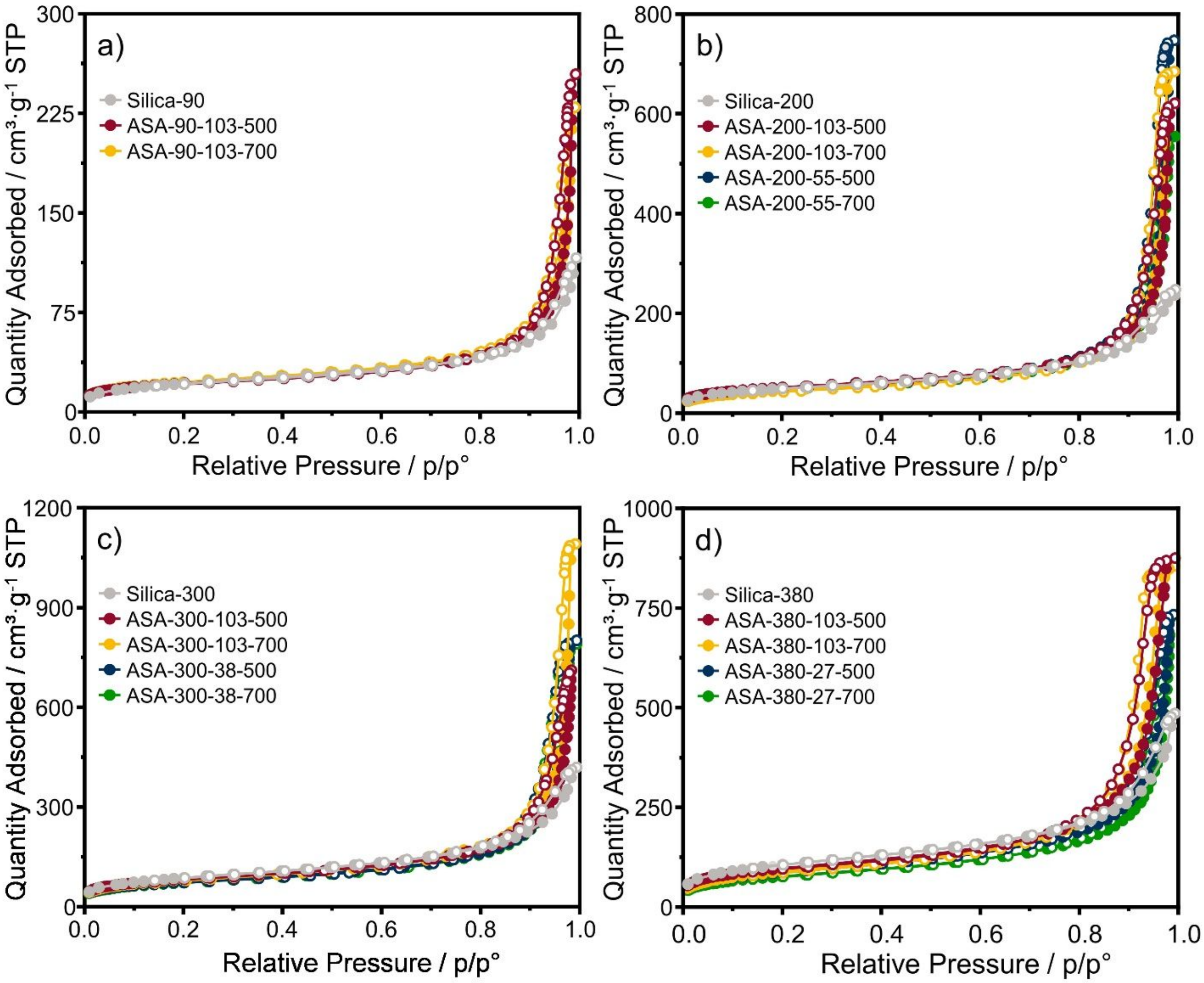

Figure S1. Adsorption (solid) and desorption (open) branches of the N_2 physisorption isotherms of a) SiO_2-90, b) SiO_2-200, c) SiO_2-300, and d) SiO_2-380.

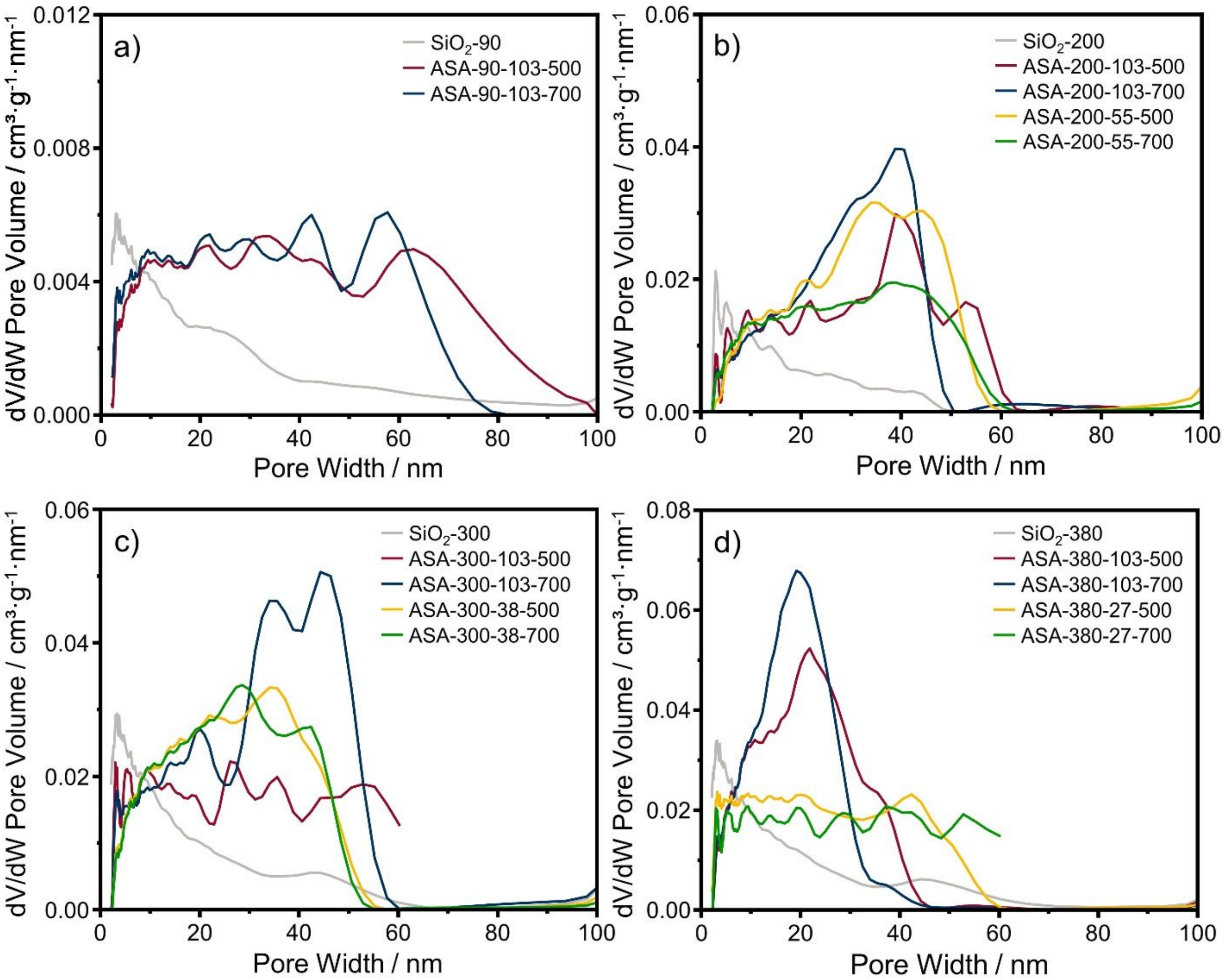

Figure S2. Pore size distributions obtained by the NLDFT approach of a) SiO_2-90, b) SiO_2-200, c) SiO_2-300, and d) SiO_2-380.

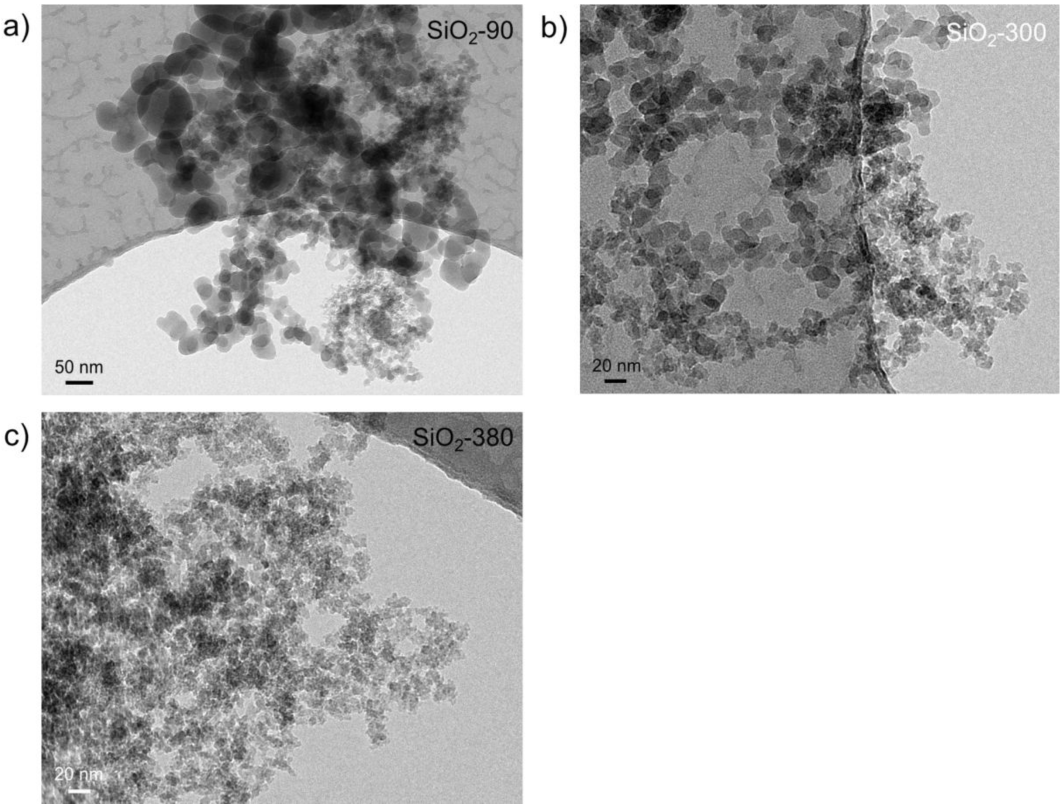

Figure S3. TEM images of a) SiO_2-90, b) SiO_2-300, and c) SiO_2-380.

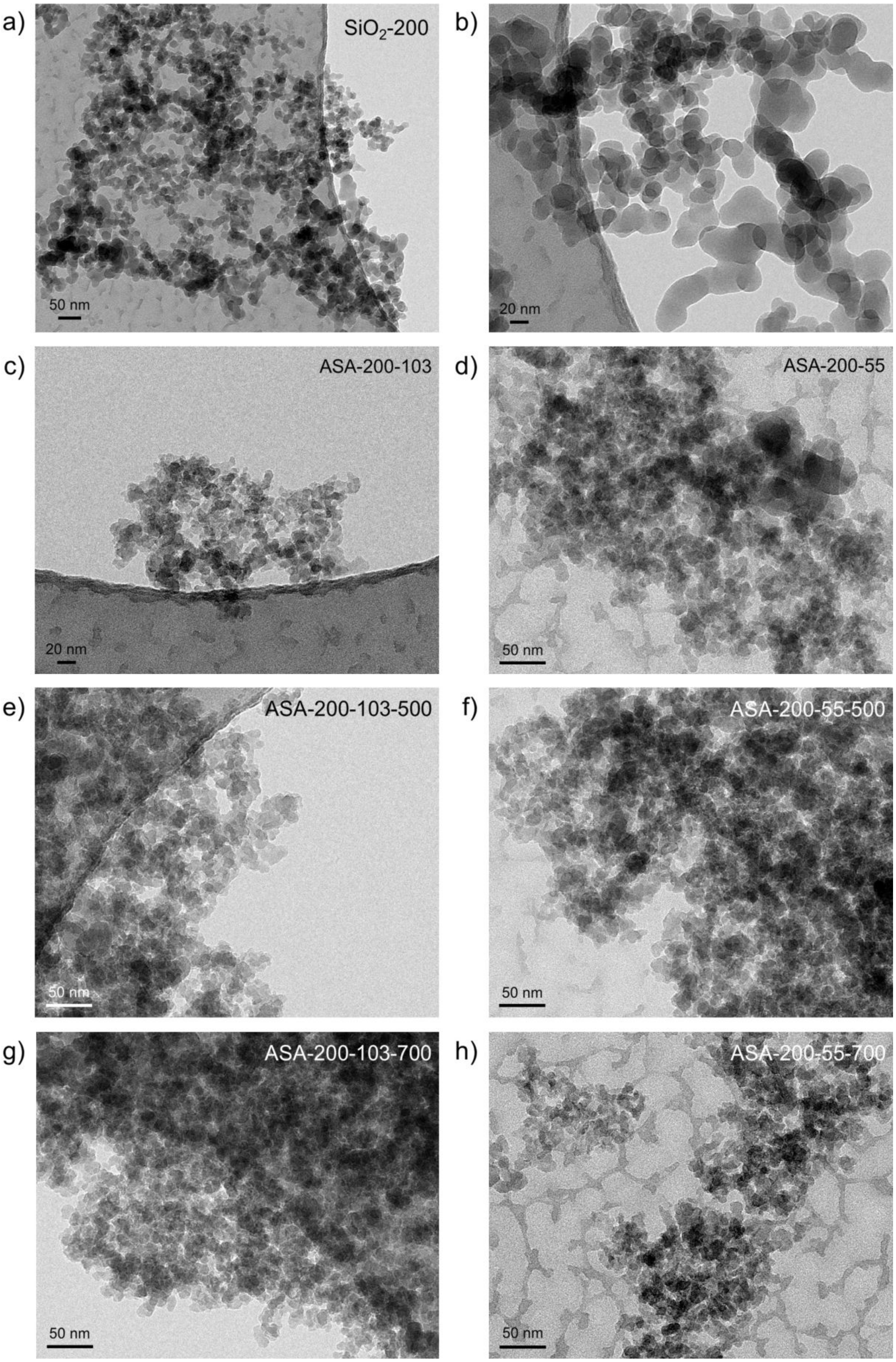

Figure S4. Representative TEM images of a, b) SiO_2-200, c) ASA-200-103, d) ASA-200-55, e) ASA-200-103-500, f) ASA-200-55-500, g) ASA-200-103-700, and h) ASA-200-55-700.

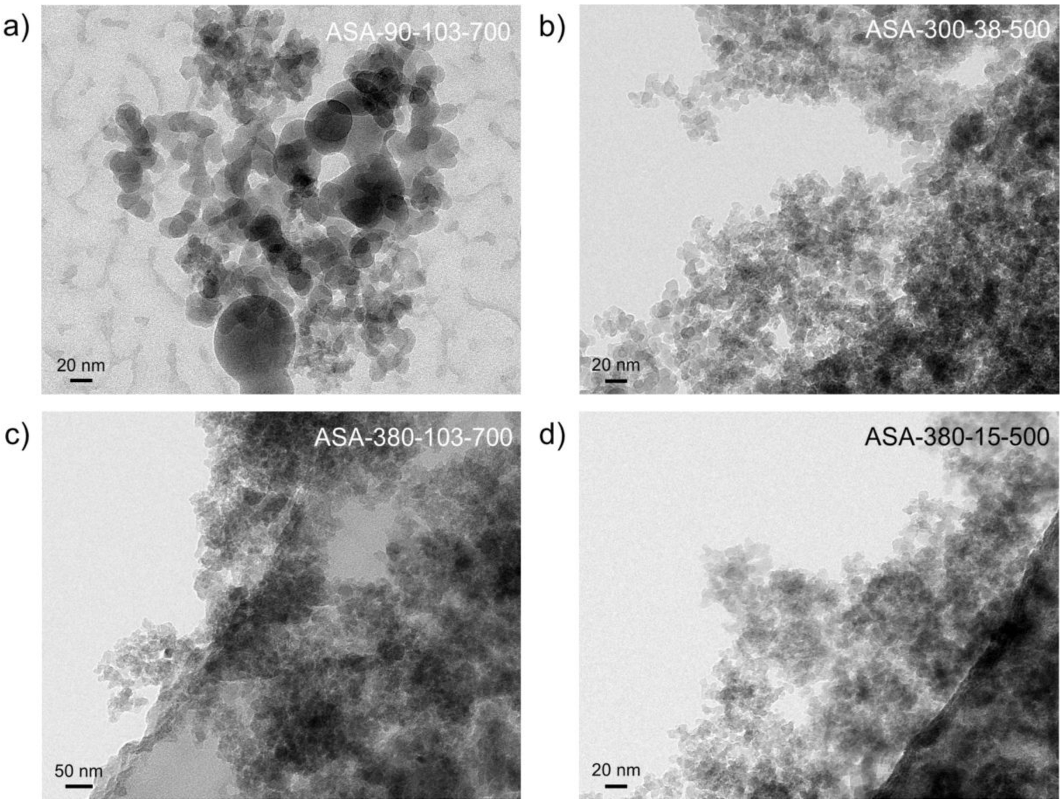

Figure S5. TEM images of a) 90-103-700, b) 300-38-500, c) 380-103-700, and d) 380-15-500.

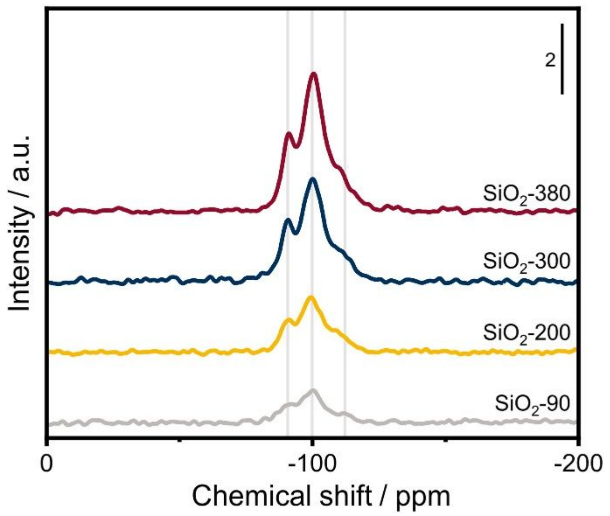

Figure S6. ^{29}Si CP MAS NMR spectra of hydrated silica samples.

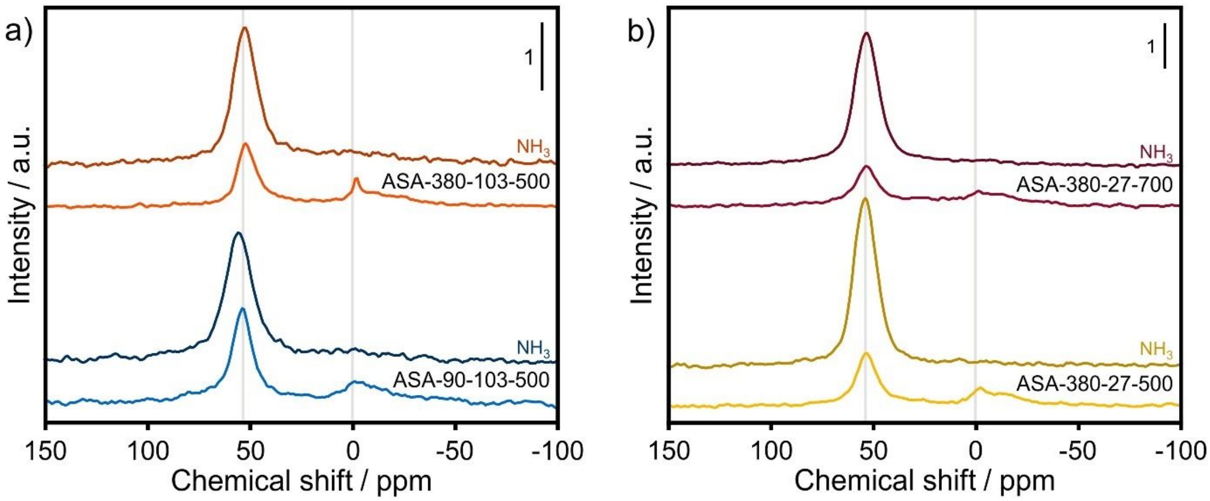

Figure S7. ^{27}Al MAS NMR spectra (a) hydrated and NH_3-treated ASA with Si/Al of 103 (8192 scans), (b) hydrated and NH_3-treated ASA-380-27 calcined at 500 and 700˚C (4096 scans).

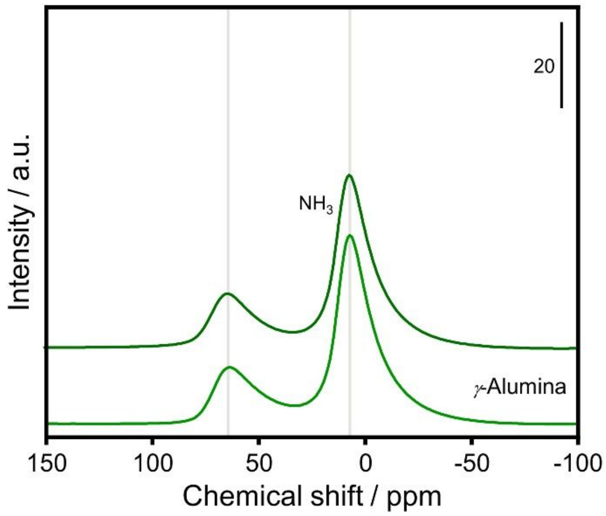

Figure S8. ^{27}Al MAS NMR spectra of γ-alumina and NH_3-treated γ-alumina.

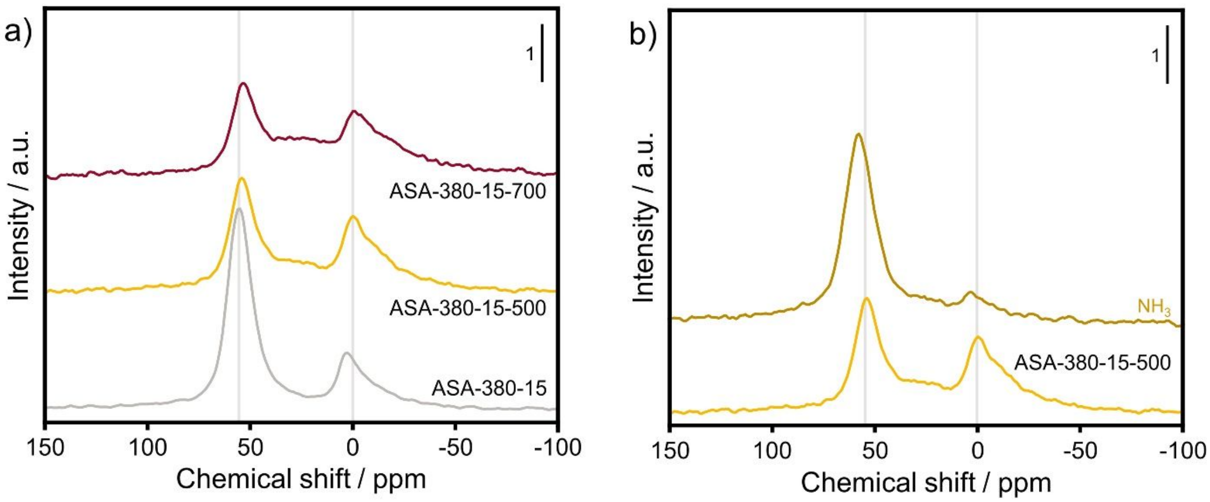

Figure S9. ^{27}Al MAS NMR spectra of a) ASA-380-15, ASA-380-15-500, ASA-380-15-700 and b) hydrated and NH_3-treated ASA-380-15-500.

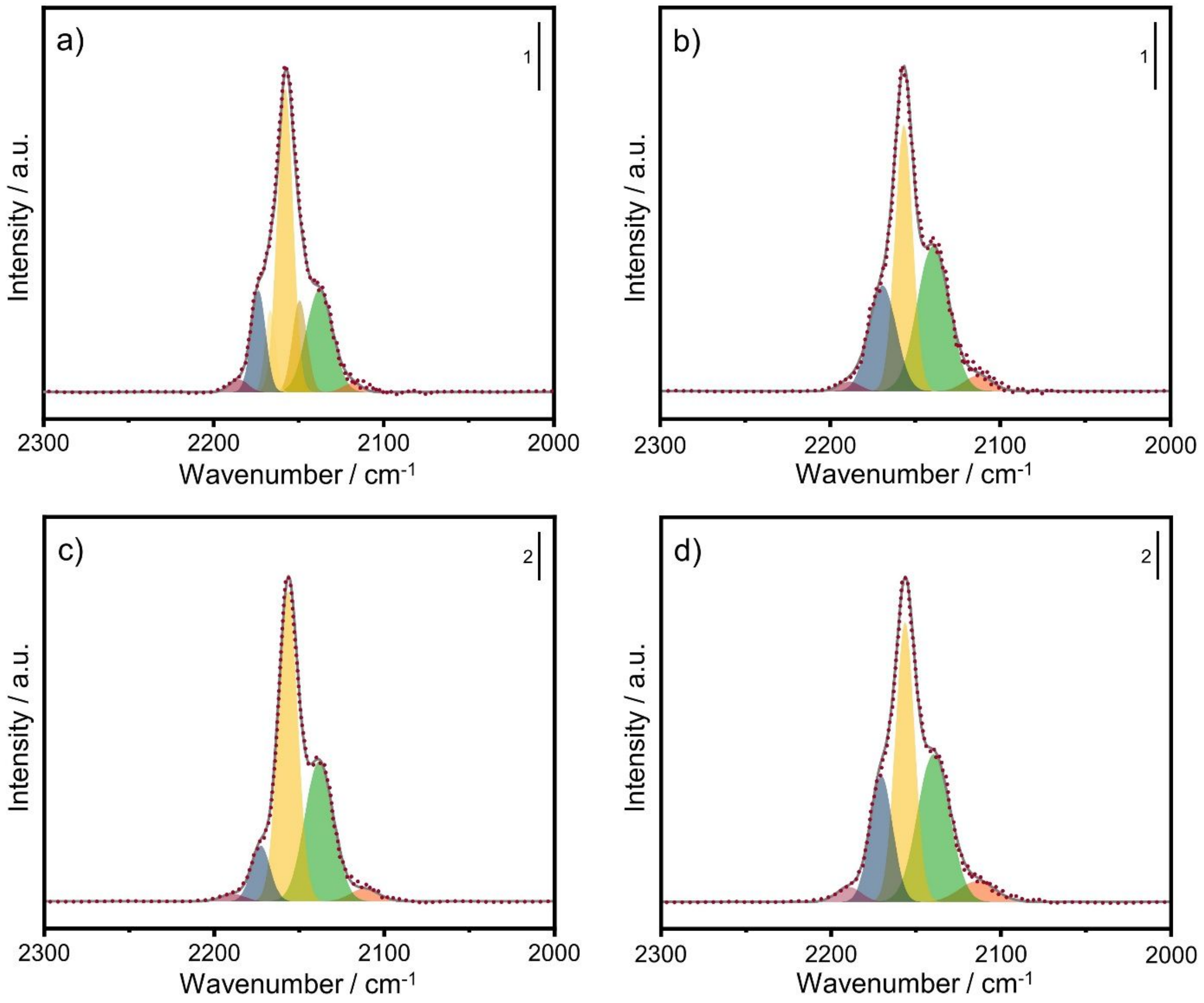

Figure S10. Peak deconvolution of the IR spectra upon CO saturation of a) ASA-90-103-700, b) ASA-200-55-700, c) ASA-380-103-500, and d) ASA-380-27-500 (red: LAS, blue: BAS, yellow: silanols, green: physisorbed CO, orange: residual physisorbed CO).

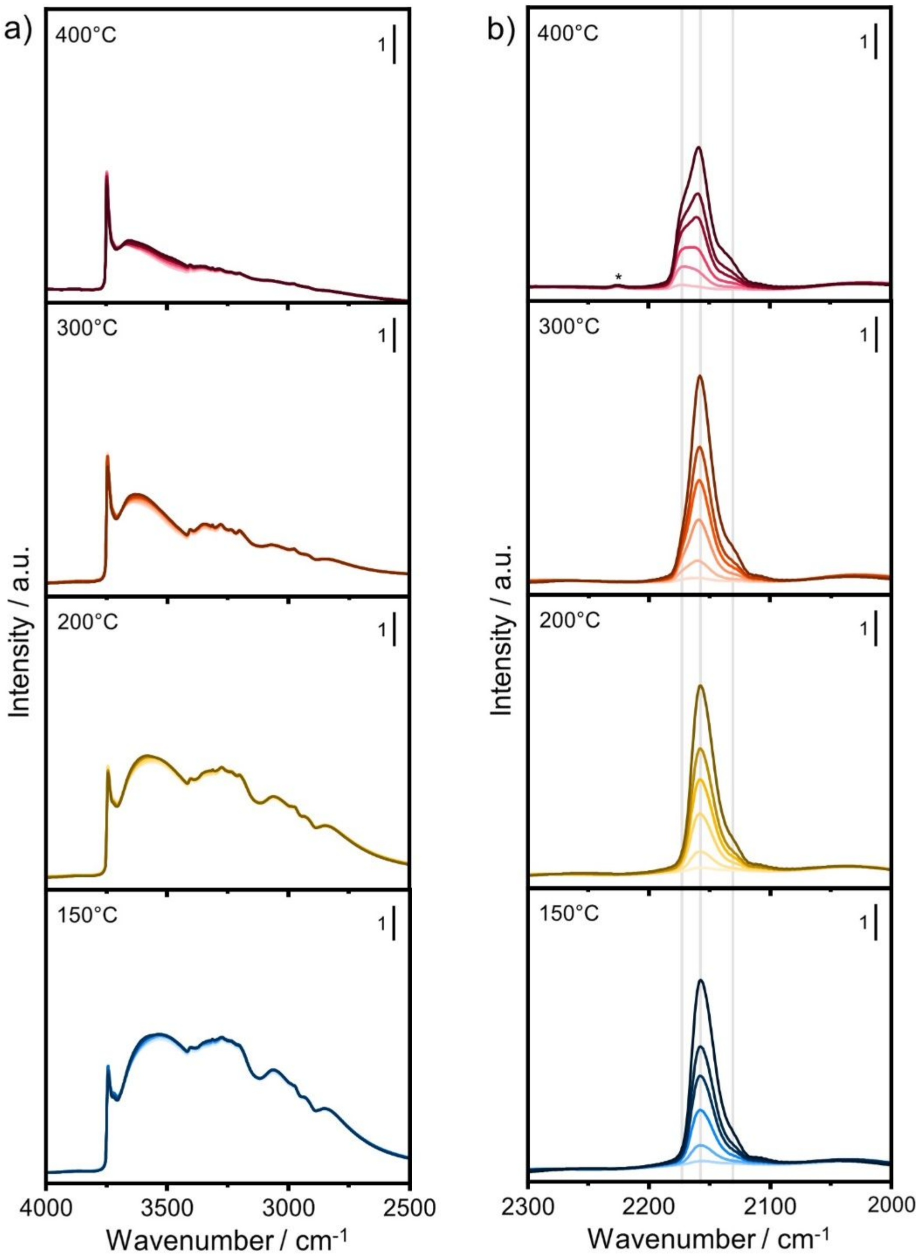

Figure S11. IR spectra of (a) the hydroxyl and (b) the carbonyl stretch region of IR spectra of 380-27 upon dosing CO at -183˚C after calcination at increasing temperatures (20 vol% O_2 in He, 1 h).

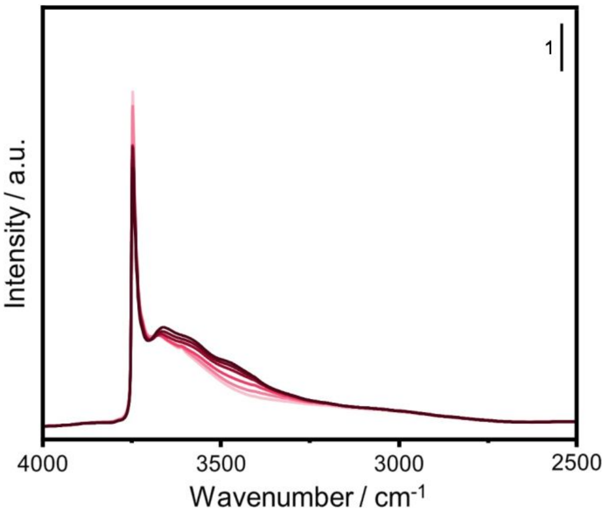

Figure S12. Hydroxyl region of IR spectra of ASA-380-27-500 as a function of the CO coverage.

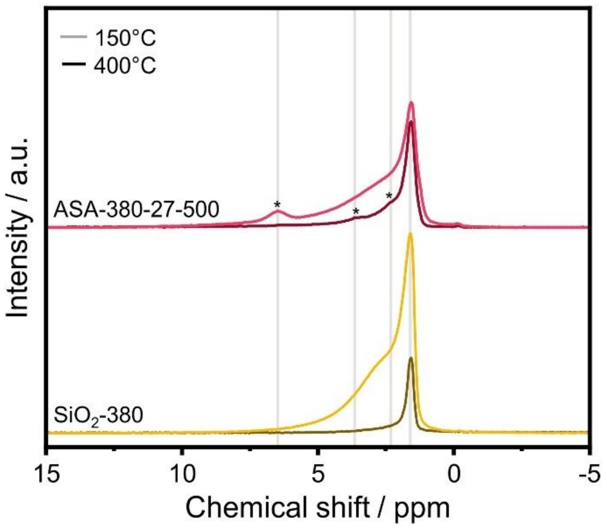

Figure S13. ^{1}H Hahn-echo MAS NMR spectra of SiO_2-380 and ASA-380-27-500.

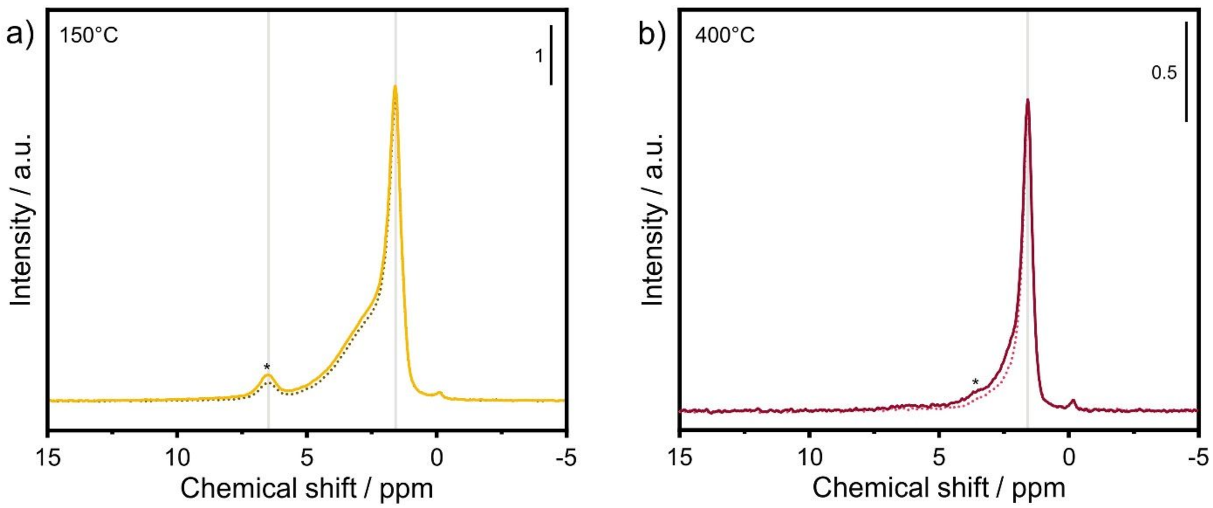

Figure S14. ^{1}H-^{27}Al TRAPDOR measurements of ASA-380-27-500 after dehydration at a) 150˚C under vacuum for 1 h and b) at 400˚C in a He/O_2 flow (33 %) for 2 h: solid and dotted lines are spectra without and with ^{27}Al irradiation.

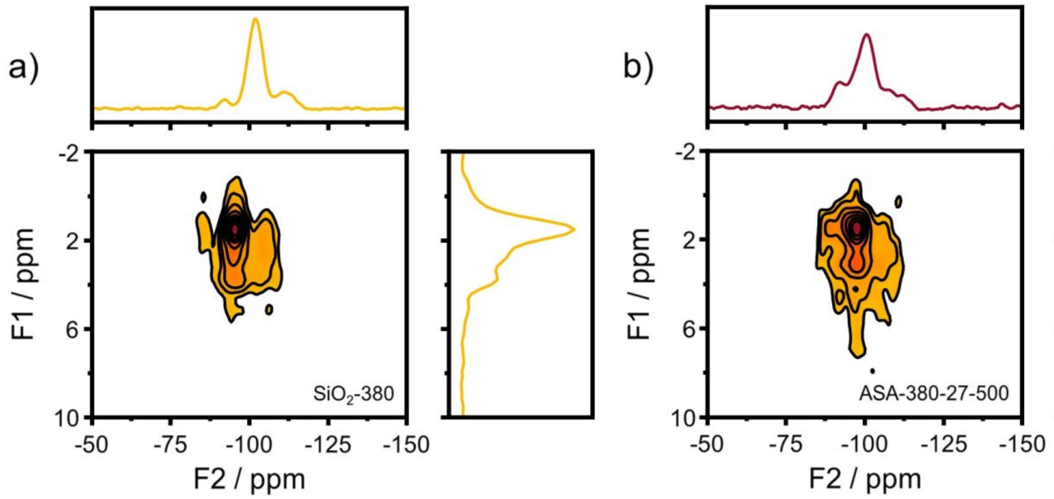

Figure S15. ^{1}H-^{29}Si CP MAS NMR of (a) SiO_2-380 and (b) ASA-380-27-500. The samples were evacuated under vacuum for 1 h at 150˚C. The 1D spectra are projections of the ^{29}Si chemical shift (F2) and the ^{1}H shift (F1).

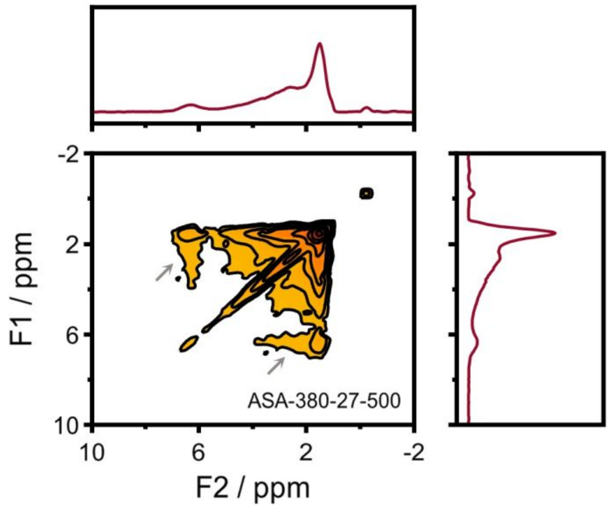

Figure S16. ^{1}H-^{1}H CP MAS NMR of ASA-380-27-500 pretreated under vacuum for 1 h at 150˚C. The 1D spectra are projections of the corresponding ^{1}H chemical shifts.

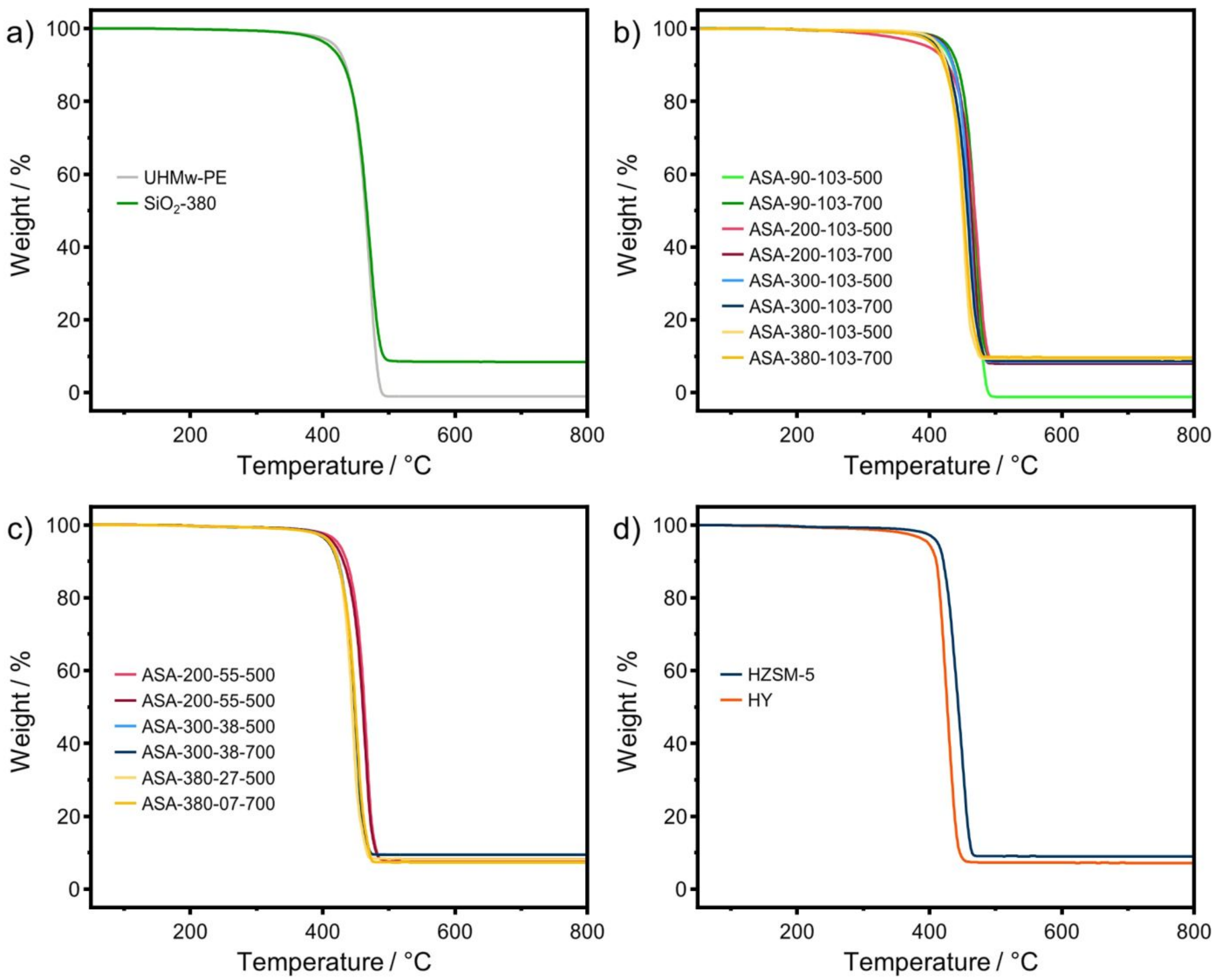

Figure S17. TGA curves catalytic pyrolysis of UHMw-PE in the a) absence or in the presence of SiO2-380, b) in the presence of ASA with equal Al loading (Si/Al ≈ 103), c) in the presence of ASA with optimal Al loading, and d) in the presence of zeolites (conditions: 5 mg polymer, 0.5 mg catalyst, 150 ml·min^{-1} He, temperature range 50-800˚C, rate 20˚C·min^{-1}).

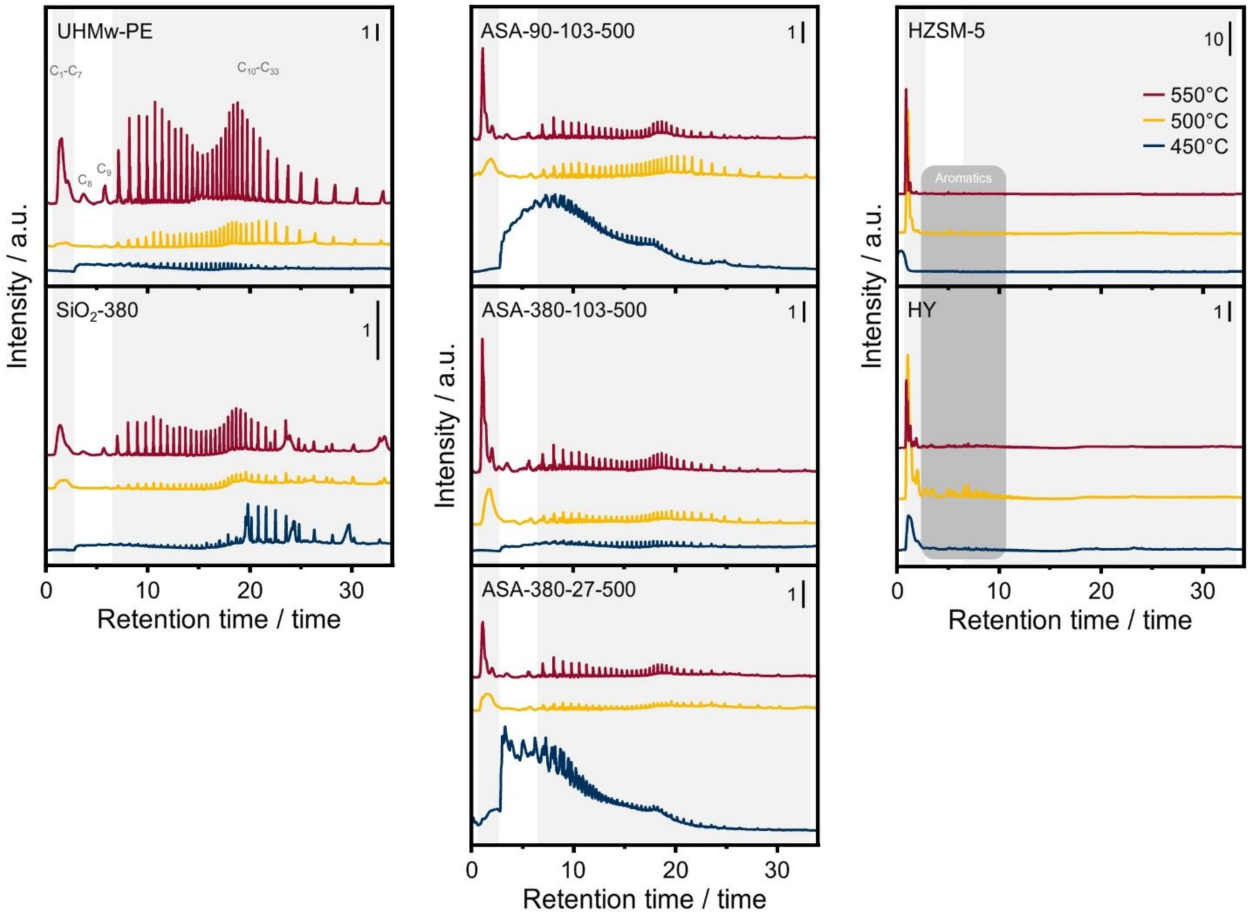

Figure S18. Pyrolysis GC-MS experiments of UHMw-PE using a Shimadzu GCMS-QP5050A equipped with a pyrolizer injector (Shimadzu, Pyr-4A).

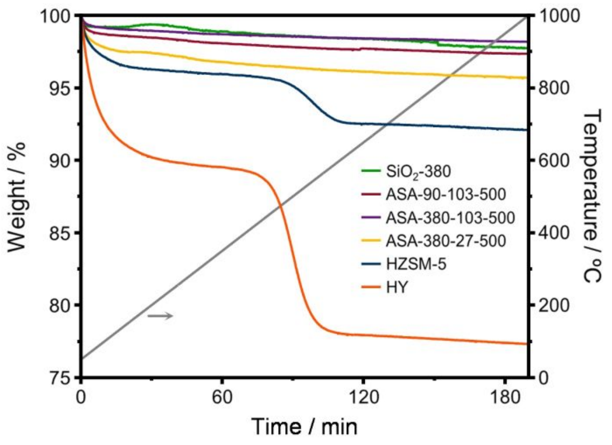

Figure S19. TGA curves of spent samples after catalytic pyrolysis of UHMw-PE (conditions: 33% O_2 in He, temperature range 50-1000˚C, rate 5˚C·min^{-1}).
